# Facile single-stranded DNA sequencing of human plasma DNA via thermostable group II intron reverse transcriptase template switching

**DOI:** 10.1038/s41598-017-09064-w

**Published:** 2017-08-21

**Authors:** Douglas C. Wu, Alan M. Lambowitz

**Affiliations:** 1 Institute for Cellular and Molecular Biology, University of Texas at Austin, Austin, Texas 78712 USA; 2 Department of Molecular Biosciences, University of Texas at Austin, Austin, Texas 78712 USA

## Abstract

High-throughput single-stranded DNA sequencing (ssDNA-seq) of cell-free DNA from plasma and other bodily fluids is a powerful method for non-invasive prenatal testing, and diagnosis of cancers and other diseases. Here, we developed a facile ssDNA-seq method, which exploits a novel template-switching activity of thermostable group II intron reverse transcriptases (TGIRTs) for DNA-seq library construction. This activity enables TGIRT enzymes to initiate DNA synthesis directly at the 3′ end of a DNA strand while simultaneously attaching a DNA-seq adapter without end repair, tailing, or ligation. Initial experiments using this method to sequence *E. coli* genomic DNA showed that the TGIRT enzyme has surprisingly robust DNA polymerase activity. Further experiments showed that TGIRT-seq of plasma DNA from a healthy individual enables analysis of nucleosome positioning, transcription factor-binding sites, DNA methylation sites, and tissues-of-origin comparably to established methods, but with a simpler workflow that captures precise DNA ends.

## Introduction

High-throughput DNA sequencing (DNA-seq) of cell-free DNA (cfDNA) in plasma and other bodily fluids has emerged as a powerful method for non-invasive prenatal testing and diagnosis of cancer and other diseases^1–3^. In healthy individuals, cfDNA in human plasma consists largely of ~167-bp DNA fragments derived from nucleosomes released by apoptosis of lymphoid and myeloid cells in blood^1,4–6^. By contrast, in a variety of pathological conditions, plasma is enriched in DNA fragments released from dying cells in the affected tissues, which can be identified by tissue-specific differences in nucleosome positioning, transcription factor occupancy, and DNA methylation sites, thereby providing diagnostic information^6,7^. Proof-of-principle experiments have demonstrated the efficacy of using cfDNA to monitor the progression of diseases, such as cancer, type I diabetes, and multiple sclerosis, as well as brain damage and transplant rejection^7–10^. In cancer patients, a significant proportion of cfDNA (7–65% in one study depending on the type of cancer) originates from tumor cells and retains epigenetic features of the tumor tissue^6,11,12^. Recent studies have shown that targeted DNA-seq of tumor-specific mutations in cfDNA in plasma can predict therapeutic response and recurrence of disease^13–16^.

As cfDNA is highly fragmented, short-read DNA-seq platforms, such as Illumina sequencing, are ideal for its analysis. Conventional double-stranded (ds) DNA-seq methods, which involve the repair of DNA ends and nicks followed by ligation of dsDNA adapters, have been used to identify single-nucleotide mutations, chromosomal rearrangements, copy number variations, and viral infections^1,2,9,17,18^. However, dsDNA-seq library preparation methods can lead to loss of damaged or short dsDNA fragments and do not capture the termini of short (40–80 nt) ssDNA fragments, which result from nicking of dsDNA by enzymes such as DNase I in regions not protected by proteins^6,19^. Recent ssDNA-seq of cfDNA in human plasma showed that mapping of these nicks yields footprints of transcription factors and other DNA-binding proteins, which combined with precise analysis of DNA ends, can be used to deduce nucleosome positioning and the tissues-of-origin of cfDNA^6,20^.

Methods for ssDNA-seq were first developed for the sequencing of ancient DNA and typically involve multiple time-consuming steps, including dephosphorylation and denaturation of dsDNA fragments, ligation of a DNA-seq adapter with a primer binding site to the 3′ end of the DNA template strand, isolation of ligated DNA (*e.g*., by using biotin-magnetic beads), copying of the DNA template strand by a DNA polymerase, end repair, and ligation of double-stranded DNA-seq adapters to the final products^6,20–22^. One commercial method (DNA SMART ChIP-Seq Kit, Clontech) differs in using a retroviral reverse transcriptase (RT; SMARTScribe) to copy the DNA template strand. In this method, the 3′ end of the template DNA strand is first tailed with poly(dT), enabling the retroviral RT to initiate DNA synthesis from an annealed poly(dA) primer. After completion of DNA synthesis, the RT template switches to an acceptor DNA oligonucleotide thereby adding a second primer-binding site for PCR and DNA-seq adapter addition. This method is relatively rapid, but poly(dT) tailing and variable non-templated nucleotide addition by the retroviral RT to the 3′ end of the DNA product prior to template switching make it difficult to precisely identify the DNA template ends and can introduce strong bias for regions with long poly(dA) or poly(dT) runs^23^. A recently developed commercial kit (ACCEL-NGS 1 S PLUS, Swift Biosciences) with a processing time of 2 h up to the PCR amplification step utilizes a proprietary adaptase technology to repair, tail, and attach a DNA-seq adapter to the 3′ end of a DNA strand. After extension of the primer annealed to the first adapter, a dsDNA adapter is ligated to the other end of the copied DNA, and the libraries are amplified with sequencing platform-specific primers. Even this simpler method employs three different enzymatic steps, leaves a low-complexity, variable length tail at one end of the DNA, an obstacle for bioinformatics analysis, and has a relatively high cost (Swift Biosciences product literature).

Recently, we developed a new method for strand-specific RNA-seq that exploits the novel properties of thermostable group II intron-encoded reverse transcriptases (TGIRTs) from bacterial thermophiles^24–26^. Group II intron RTs function in the mobility of bacterial and organellar retrotransposons called mobile group II introns by a mechanism (“retrohoming”) that requires synthesis of a full-length cDNA from a highly structured group II intron RNA with high fidelity and processivity, properties that are useful for RNA-seq.^27^. After overcoming long-standing difficulties with the purification of highly active group II intron RTs in soluble form, we showed that two TGIRT enzymes from bacterial thermophiles have higher processivity and fidelity than the retroviral RTs commonly used for RNA-seq, as well as a novel template-switching activity that is minimally dependent upon base pairing and enables RNA-seq adapter addition without using RNA ligase^24,25^. Taking advantage of these properties, we showed that TGIRT enzymes enable the construction of comprehensive RNA-seq libraries from small amounts of starting material in ~2 h up to the PCR amplification step^25^. Validation of the method by Illumina sequencing of ribodepleted, fragmented human reference RNAs showed significantly better coverage of 5′-proximal regions of mRNAs and splice junctions, better quantitation of RNA spike-ins, and higher strand specificity than the widely used TruSeq v3 method^26^. During these studies, we found that TGIRT enzymes could also template-switch to and copy DNA oligonucleotides and cfDNAs in human plasma, raising the possibility that an analogous method could be developed for ssDNA-seq^24,25,28^.

Here, we used TGIRT template-switching to develop a streamlined ssDNA-seq protocol, which enables construction of DNA-seq libraries from small amounts of starting DNA (2–2.5 ng plasma DNA in this study) in ~2 h. Initial experiments in which we used the method to sequence *E. coli* genomic DNA showed that the TGIRT enzyme has surprisingly robust DNA-dependent DNA polymerase activity for an RT, with manageable error rates and uniformity of coverage comparable to Nextera-XT. We then showed that TGIRT-seq of human plasma DNA enables the analysis of nucleosome positioning, transcription factor-binding sites, DNA methylation sites, and tissues-of-origin comparably to conventional methods, but with a simpler workflow that captures precise DNA ends. We anticipate that TGIRT-seq could be used in place of conventional, more cumbersome and/or expensive ssDNA-seq methods for diagnostic applications requiring the sequencing of highly fragmented DNAs, such as cfDNAs or DNAs from formalin-fixed paraffin-embedded (FFPE) tumor samples.

## Results

### Overview of the method

**Figure 1:**
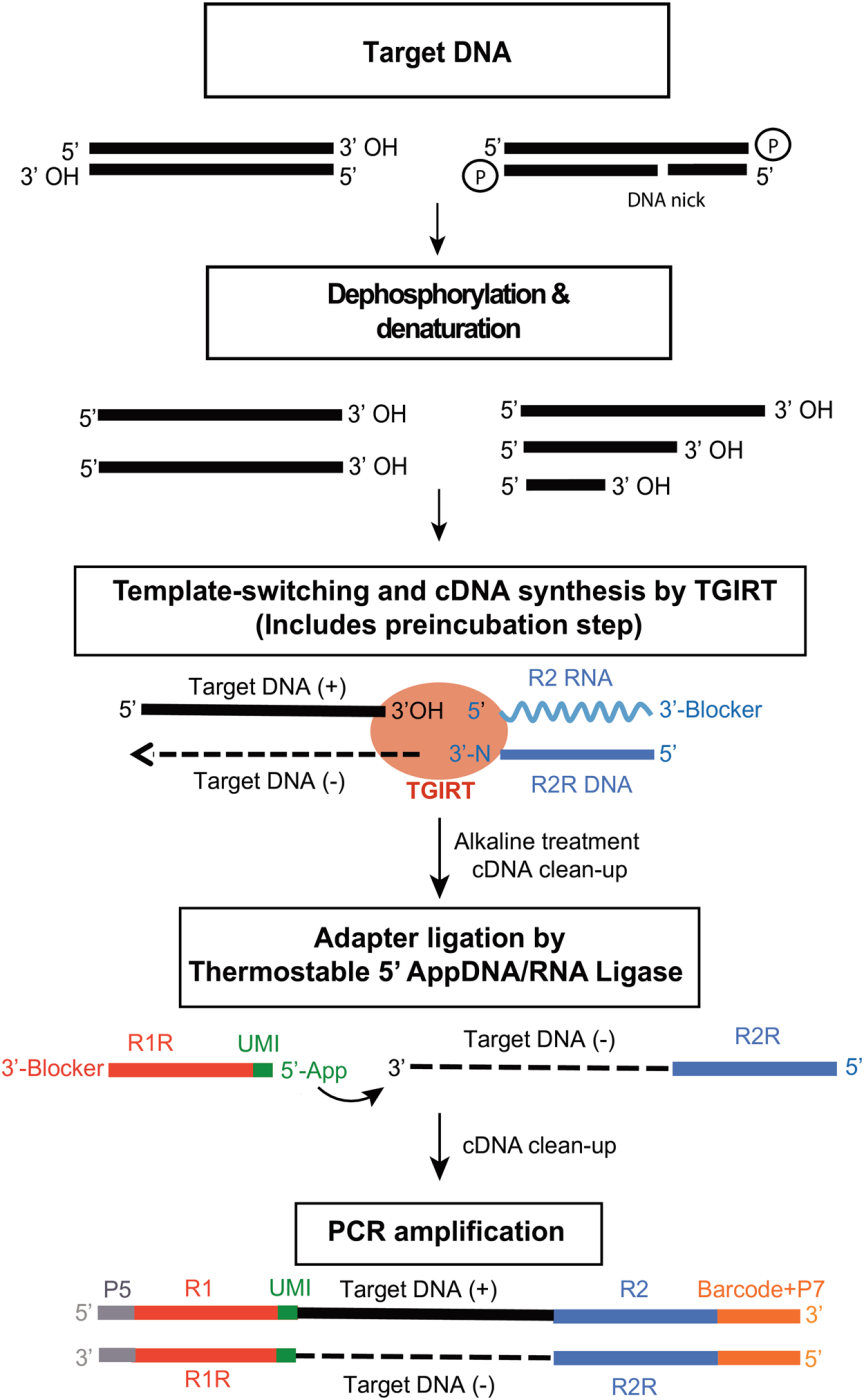
TGIRT ssDNA-seq workflow. The target DNA (2–50 ng in this work) is treated with alkaline phosphatase to remove 3′ phosphates and heat denatured prior to DNA-seq library construction. The resulting ssDNAs with 3′ OH termini are then used for TGIRT template-switching DNA synthesis coupled to Illumina read 2 reverse (R2R) DNA-seq adapter addition. In this novel reaction, the TGIRT-III enzyme (InGex) binds first to a synthetic 34-bp R2 RNA/R2R DNA heteroduplex in which the R2R DNA primer has a single nucleotide 3′ overhang that can direct TGIRT template switching by base pairing to the 3′ nucleotide of a target DNA strand. For minimally biased library preparation, the 3′-overhang nucleotide is an equimolar mixture of A, C, G, and T (denoted N). A 3′-blocking group (C3 spacer; 3′SpC3) is attached to the end of the R2 RNA oligonucleotide to prevent template-switching to that RNA. After the TGIRT enzyme extends the DNA primer to produce a DNA copy of the target DNA strand with an R2R adapter seamlessly linked to its 5′ end, a 5′ adenylated (App) Illumina read 1 reverse (R1R) adapter is added to its 3′ end by single-stranded DNA ligation using a Thermostable 5′ AppDNA/RNA ligase (New England Biolabs). In the workflow shown, an unique molecular identifier (UMI) is positioned at the 5′ end of the R1R DNA oligonucleotide. A final PCR step adds flow cell capture sites and barcodes for Illumina sequencing and sample multiplexing.

Figure 1 outlines the TGIRT template-switching method used here for ssDNA-seq. The method is similar to that developed previously for strand-specific RNA-seq, but with reaction conditions optimized for DNA templates (see Supplementary Figs S1 and S2). In the first step, the TGIRT enzyme (TGIRT-III, a derivative of the *Geobacillus stearothermohilus* GsI-IIC group II intron RT; InGex) binds to a short synthetic RNA template/DNA primer heteroduplex substrate in which the DNA primer contains a reverse complement of Illumina read 2 adapter sequence (R2R) and has a single-nucleotide 3′ DNA overhang (see Supplementary Table S1). The latter can direct seamless TGIRT template switching by base pairing to the 3′ nt of a target DNA strand^24^. For the preparation of minimally biased libraries, the initial template-primer substrate has an equimolar mix of A, C, G, and T (denoted N) 3′ DNA overhangs and is added in excess to the target nucleic acid. The DNA fragments to be sequenced are treated enzymatically to remove 3′ phosphates, which block TGIRT template switching, and heat denatured. After pre-incubating the enzyme with template-primer substrate, a step that significantly increases the efficiency of the DNA synthesis (see Supplementary Fig. S2), the enzyme template-primer complex is added to the DNA to be sequenced, and the reaction is initiated by adding dNTPs and incubating at 60 °C for times dictated by the length of the DNA templates. A high salt reaction medium is used as a tradeoff that decreases the efficiency of the enzyme in copying DNA templates, but also suppresses multiple template switches that could result in artificial chimeric DNA sequences (see Supplementary Fig. S1). After the completion of DNA synthesis, a second DNA-seq adapter containing a reverse complement of an Illumina read 1 adapter sequence (R1R) is ligated to the 3′ end of the DNA product strand by an efficient single-stranded DNA ligation using thermostable 5′ AppDNA/RNA ligase (New England Biolabs), and finally the target DNA sequences are amplified by PCR using primers that introduce Illumina flow cell capture sites and sample barcodes for sequencing. An unique molecular identifier (UMI), a randomized nucleotide sequence that tags individual DNA products, can be incorporated into either the 5′ or 3′ adapter (shown in Fig. 1 for the R1R adapter, the location of the UMI in the present work).

### TGIRT ssDNA-seq metrics

To systematically evaluate how well the TGIRT-III enzyme copies DNA, we carried out TGIRT-seq of *E. coli* K12 (MG1655) genomic DNA and compared the resulting datasets to those obtained previously for this DNA using Nextera-XT, a widely used double-stranded DNA-seq method. For this test, the *E. coli* DNA was fragmented by sonication (Covaris) to an average size of ~250 bp (see Supplementary Fig. S3), and DNA-seq libraries were prepared by using the TGIRT-seq workflow of Fig. 1, with a 13-nt UMI at the 5′ end of the R1R oligonucleotide (RIR-UMI; see Supplementary Table S1). Ligation of the R1R oligonucleotide with an UMI at its 5′ end or with an UMI preceded by a short fixed sequence (used below) was more efficient than ligation of an R1R oliognucleotide without an UMI (Wilcoxon-Mann-Whitney test p-values = 0.002 and <0.001, respectively; see Supplementary Fig. S4) and may also decrease ligation bias. The libraries were sequenced on an Illumina NextSeq 500 instrument to obtain ~4 million 2 × 75 nt reads (~130X coverage of the *E. coli* genome). Mapping statistics for three technical replicates for TGIRT-seq (datasets EG1–3) compared to three technical replicates for Nextera-XT (NX10, 50, 60; also sequenced on a NextSeq 500; Illumina Basespace Project 21071065)^29^ are summarized in Supplementary Table S2. The proportion of chimeric reads, which could potentially result from multiple template switches, was 1–2 × 10^−5^, comparable to that in the Nextera-XT datasets (1–3 × 10^−5^).

**Figure 2:**
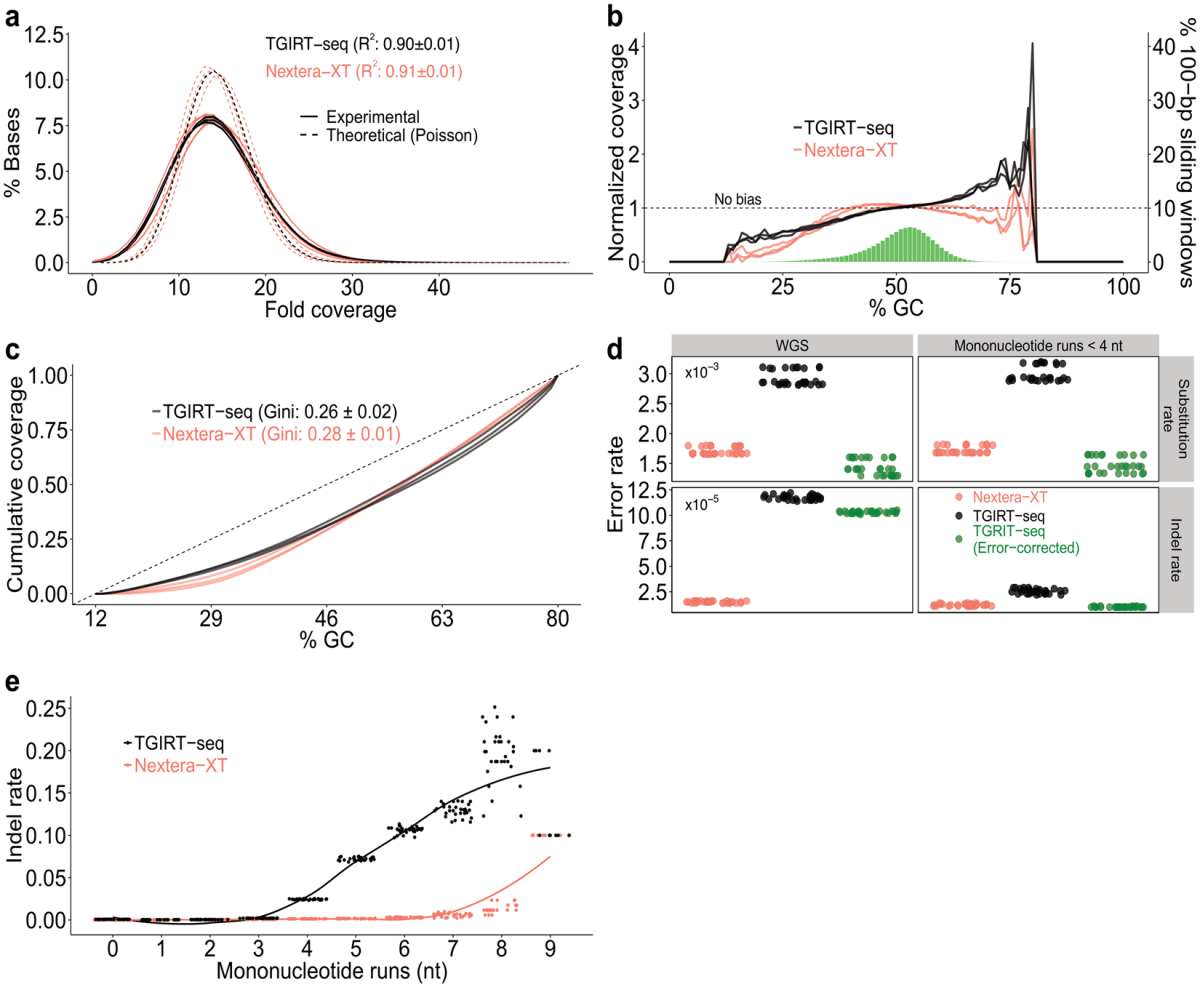
TGIRT DNA-seq metrics. *E. coli* K12 strain MG1655 genomic DNA was sequenced by TGIRT-seq (3 replicates with R1R-UMI), and the resulting datasets (EG1–3; Supplementary Table S2) were compared to datasets obtained previously for this DNA by Nextera-XT (3 replicates; Illumina Basespace Project 21071065; datasets NX10, 50, 60). (**a**) Histogram of base coverage across the *E. coli* genome in 10 randomly subsampled datasets of ~16X coverage from each TGIRT-seq (black) and Nextera-XT (pink) replicate. The TGIRT-seq and Nextera-XT subsampled datasets show similar agreement to expected Poisson distributions (dashed black and pink lines; R^2^ = 0.90 ± 0.01 and 0.91 ± 0.01, respectively). (**b**) Normalized coverage as a function of GC content over a 100-nt sliding window across the *E. coli* genome for TGIRT-seq (black lines; 3 replicates) and Nextera-XT (pink lines; 3 replicates) compared to theoretical uniform coverage (dashed line). The green histogram shows the percentage of windows of each GC content in the *E. coli* genome. TGIRT-seq gives better coverage of regions having low GC content, but over-represents regions of GC content >60%. **(c)** Lorenz curves showing the cumulative distribution of normalized coverage in a 100-nt sliding window across the *E. coli* genome for TGIRT-seq and Nextera-XT (3 replicates each). The curves show that TGIRT-seq and Nextera-XT give cumulatively similar uniform coverage over the range of GC contents in the *E. coli* genome (Gini coefficients: 0.26 ± 0.02 and 0.28 ± 0.01, respectively). Dashed line denotes no bias. **(d)** Base substitution and indel rates for TGIRT-seq with or without UMI correction (black and green dots, respectively) compared to Nextera-XT (pink dots) for 10 subsampled datasets at 16X coverage from each TGIRT-seq and Nextera-XT replicate. The left panels show error rates for the whole genome (WGS), and the right panels show the error rates excluding mononucleotide runs ≥4. **(e)** Plot of indel frequency versus mononucleotide run length for 10 subsampled libraries at 16X coverage from each of 3 error-corrected TGIRT-seq (black) and 3 Nextera-XT (pink) datasets. TGIRT shows an increase in indel frequency at mononucleotide runs ≥ 4 (see also Supplementary Fig. S7).

Whole-genome DNA-seq with no sampling bias should ideally give uniform read coverage for each base in the genome^30^. To compare the uniformity of coverage of TGIRT-seq to that of Nextera-XT, we determined the fold coverage of each base in the *E. coli* genome at a fixed sequencing depth relative to that expected for a theoretical Poisson distribution (Fig. 2a). To normalize sequencing depth of the TGIRT-seq and Nextera-XT datasets, we compared 10 randomly subsampled libraries at 16X coverage (limited by the sequencing depth of the Nextera-XT datasets) from each technical replicate obtained by each method (3 for TGIRT-seq and 3 for Nextera-XT). The plot shows that TGIRT-seq and Nextera-XT both give relatively uniform coverage of the genome, with similar deviations from the expected Poisson distributions for the subsampled datasets (R^2^ = 0.90 and 0.91, respectively; Wilcoxon-Mann-Whitney test p-value = 1 × 10^−4^; F-test p-value = 4 × 10^−5^; Fig. 2a).

To assess GC content biases in TGIRT-seq compared to Nextera-XT, we plotted both the normalized coverage as a function of GC-content over a 100-nt sliding window across the *E. coli* genome (Fig. 2b), and the cumulative distribution of normalized coverage as a function of GC content (Fig. 2c). The first plot shows that TGIRT-seq gives better representation of regions having low GC content than does Nextera-XT, but that TGIRT-seq coverage increases linearly with increasing GC content (R^2^ for linear regression: 0.95 ± 0.02 for TGIRT-seq compared to 0.58 ± 0.17 for Nextera-XT), leading to over-representation of regions of GC content >60% (Fig. 2b). The second plot, Lorenz curves of the cumulative proportion of normalized coverage as a function of GC content, provides an overall measure of coverage inequality, the Gini coefficient (the area between the Lorenz curve and the diagonal expected for uniform coverage divided by the total area under the diagonal). The plots show that TGIRT-seq and Nextera-XT give similarly uniform coverage over the range of GC contents in the *E. coli* genome (Gini coefficients = 0.26 ± 0.02 and 0.28 ± 0.01 for TGIRT-seq and Nextera-XT technical replicates, respectively; Wilcoxon-Mann-Whitney test p-value = 0.4; F-test p-value = 0.48; Fig. 2c).

The method used for DNA-seq adapter attachment can lead to non-random sampling indicated by sequence biases at the 5′- and 3′-ends of the copied DNA fragments. Analysis of these biases showed that TGIRT template-switching introduces minimal sequence bias at the 3′ end of the copied DNA fragment compared to Nextera-XT, but that R1R adapter ligation by the thermostable 5′AppDNA/RNA ligase introduces bias for C and G at the positions 2 and 3, respectively, from the 5′ end (see Supplementary Fig. S5), as reported previously^26,31,32^. The latter bias was somewhat less for the R1R-UMI adapter with the 13-nt UMI directly at its 5′ end (R1R-UMI) than for an R1R adapter with a short fixed sequence preceding the UMI (R1R-UMI + CGATG; see Supplementary Fig. S5). Simulations indicated that it is this ssDNA-ligation bias that leads to higher representation of regions of high GC content by TGIRT-seq (Fig. 2b and see Supplementary Fig. S6).

TGIRT enzymes copy RNA templates with higher fidelity than do conventional retroviral RTs^24,33,34^, but are not expected to have as high fidelity as a DNA polymerase copying a DNA template. Comparison showed that the base substitution rate for TGIRT-seq without UMI correction for errors introduced by PCR and sequencing was 1.7-fold higher than Nextera-XT, but was comparable to that for the Nextera-XT datasets after UMI correction (Fig. 2d, top left panel). The Nextera-XT datesets were obtained without an UMI, and their substitution error rate is assumed to be limited by that for Illumina sequencing (0.1–1% for NextSeq 500)^35,36^. The indel rate for TGIRT-seq on DNA was ~8-fold higher than Nextera-XT with or without UMI correction (Fig. 2d, bottom left panel). Further analysis showed that this higher indel rate for TGIRT-seq is due largely to template slippage at mononucleotides runs ≥4 nt, particularly deletions at runs of Ts and insertion of extra A residues at runs of As (Fig. 2e and see Supplementary Fig. S7). The Nextera-XT datasets show an increased frequency of indels at mononucleotide runs ≥7 nt, which may include contributions from base calling errors during Illumina sequencing (Fig. 2e)^30,37^. When mononucleotide runs ≥4 nt were excluded from the analysis, the substitution rate for TGIRT-seq was unchanged but the indel rate was substantially decreased and comparable to that of Nextera-XT.

In the TGIRT ssDNA-seq method used here, UMIs are added by single-stranded DNA ligation of the R1R primer to the copied DNA strand, and UMI re-sampling would occur if TGIRT-seq recycles a DNA template after the completion of DNA synthesis so that the same template has different UMIs. To assess the level of UMI resampling, we compared the percentages of duplicate DNA fragments having identical 5′ and 3′ termini but different UMIs in the TGIRT-seq datasets to those in simulated datasets generated by randomly selecting the same length *E. coli* sequences with TGIRT-seq 5′ and 3′ end biases (see Supplementary Fig. S6). The simulated datasets enabled us to estimate the number of duplicate 5′ and 3′ termini expected to result from fragmentation and ligation biases (see Supplementary Fig. S8). Using a Chi-square test, we found that the low frequency of templates with the same coordinates and different UMIs (<1%) can be accounted for by DNA fragmentation and ligation biases rather than UMI resampling, which appears to be minimal for TGIRT-seq. Overall, the analysis of the DNA-seq metrics shows that the TGIRT-III enzyme performs surprisingly well for an RT copying DNA templates and suggest that this will not be a major limitation for most DNA-seq analysis applications.

### TGIRT-seq of cfDNA in human plasma

To assess its clinical utility, we used TGIRT-ssDNA-seq to sequence cfDNA from plasma of a healthy male individual and compared the resulting datasets to those obtained in a recent study using a conventional ssDNA-seq method^6^. In these experiments, TGIRT ssDNA-seq libraries were constructed from 2–2.5 ng of plasma cell-free (cf) DNA by using the workflow of Fig. 1. Bioanalyzer traces showed that the purified cfDNA has a peak at ~170 nt, the size expected for DNA fragments protected in chromatosomes (nucleosome cores plus linker histone H1; see Supplementary Fig. S9)^1,6,19^, and the final libraries show a peak of ~300 bp corresponding to the 170-bp input DNA with added Illumina flow cell adapters, sequencing adapters, and the 13-nt UMI (see Supplementary Fig. S9). The libraries were sequenced on an Illumina NextSeq 500 to obtain 50–120 million 2 × 75 nt reads, corresponding to ~2.5–5X coverage of the human genome. Mapping statistics for nine TGIRT-seq replicates (six obtained with the UMI directly at the 5′ end of the R1R adapter and three with the UMI preceded by a short fixed sequence) compared to the published datasets for conventional ssDNA-seq of plasma DNA from healthy male and female individuals are summarized in Supplementary Table S3.

**Figure 3:**
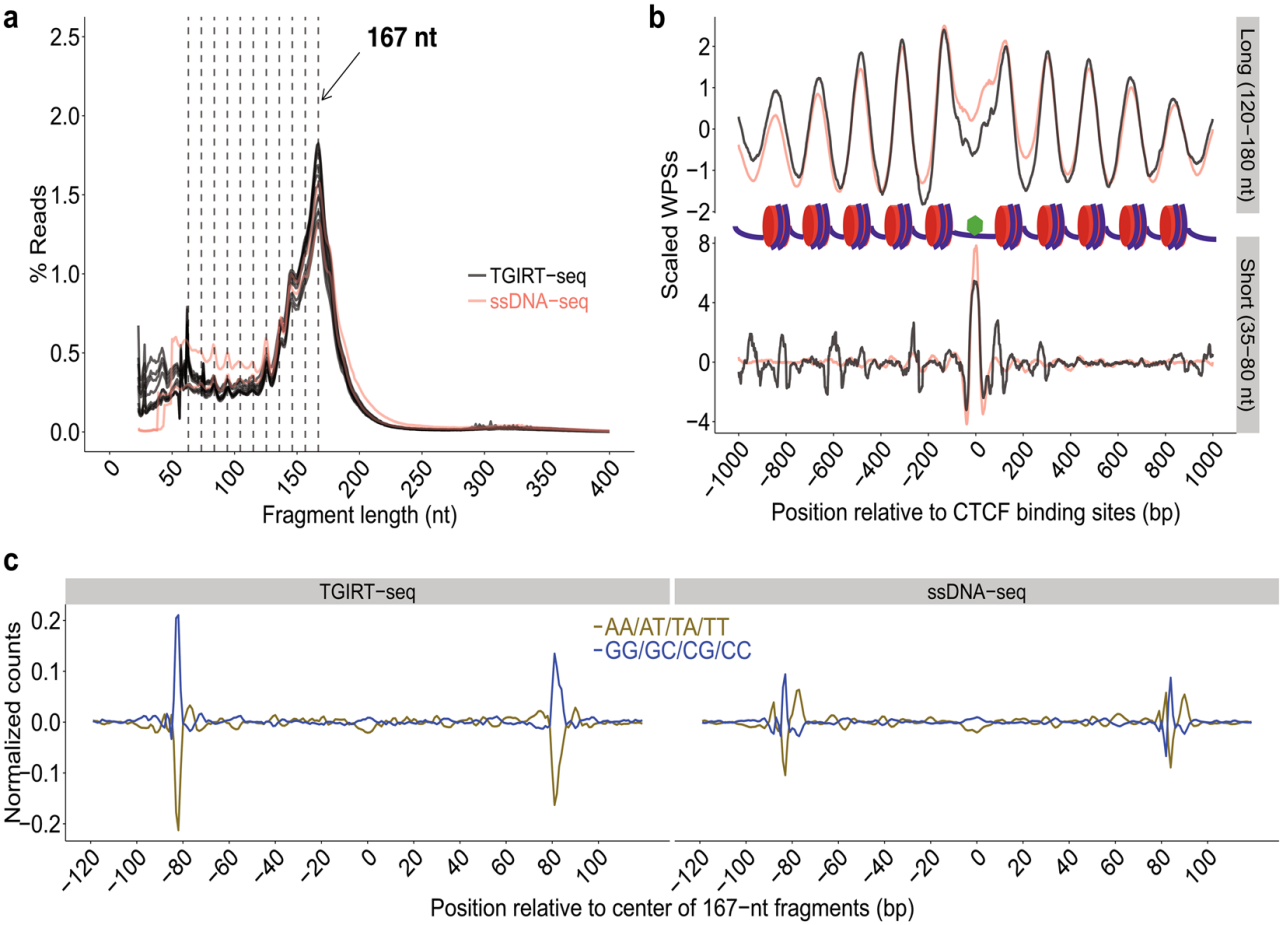
TGIRT ssDNA-seq of human plasma DNA enables analysis of nucleosome positioning and transcription factor-binding sites. Plasma DNA from a healthy male individual was sequenced by TGIRT-seq, and the resulting datasets (PD1-9; see Supplementary Table S3) were compared to those for plasma DNA from other healthy individuals sequenced by a conventional ssDNA-seq method (SRR2130051 and SRR2130052)^6^. **(a)** Fragment length distribution for TGIRT-seq (black lines; 9 datasets for a healthy male individual) and conventional ssDNA-seq (pink lines; 2 datasets, one for a male and the other for a female individual). The peaks at 167 nt reflect DNA that is protected from cleavage by endogenous DNases by packaging in nucleosomes. The vertical dashed lines indicate subpeaks with a periodicity of ~10.4 nt protected from endogenous DNase I nicking of exposed regions of dsDNA in nucleosome^6,38^. **(b)** Meta-analysis of scaled window protection scores (WPSs) within ±1 kb of all annotated binding sites for transcription factor CTCF (green hexagon) in healthy male individuals analyzed by TGIRT-seq and ssDNA-seq. TGIRT-seq (black line; combined datasets from 9 libraries) and conventional ssDNA (pink line; healthy male individual, SRR2130051) show similarly protected DNA regions both for longer DNA fragments (120–180 nt) protected in nucleosome cores and shorter DNA fragments (35–80 nt) protected by bound transcription factor CTCF (top and bottom graphs, respectively). **(c)** Dinucleotide frequencies as a function of nucleotide position across 240-bp genomic windows centered at the mid-point of 167-nt DNA fragments for TGIRT-seq (left; combined datasets for 9 replicates) and conventional ssDNA-seq (right; healthy male individual; SRR2130051). Dinucleotide frequencies were extracted from the datasets by a customized script and normalized by using a median filter with a window size of 101 nucleotides.

Analysis of the datasets showed that the TGIRT-seq faithfully reproduces the 167-nt peak profile for nucleosome-protected DNA, as well as shorter cfDNA fragments (35–80 bp) similar to conventional ssDNA-seq (Fig. 3a). This includes a superimposed cleavage pattern of 10.4-base periodicity (gray dashed lines), which corresponds to one turn of the helix and is thought to reflect endogenous DNase I cleavage in the exposed minor groove of the nucleosome bound DNA^6,38^. As in the previous ssDNA-seq analysis, which used a lengthier workflow, the 10.4-bp sub-peaks are 3-nt shorter than would be obtained by dsDNA-seq due to the ability of ssDNA-seq to faithfully detect true DNA ends^6^.

To test if TGIRT-seq captures regions of cfDNA protected from DNase digestion in plasma by nucleosome- or transcription factor-binding, we plotted window protection scores (WPSs) by calculating the number of DNA fragment ends over a sliding window within 1 kb of binding sites for the abundant transcription factor CTCF (CCCTC-binding factor), which is known to function nucleosome positioning^6^. The WPSs were scaled by zeroing the mean and dividing by the standard deviation. To match the sequencing depth of the previous ssDNA-seq analysis, we combined the datasets for all nine TGIRT-seq libraries (PD1–9; see Supplementary Table S3). The calculation of WPSs was done separately for long DNA fragments (120–180 nt) using a 120-bp sliding window and short DNA fragments (35–80 nt) using a 16-bp sliding window^6^.

As found previously by conventional ssDNA-seq, the WPSs for the long DNA fragments show a periodicity of ~180 bp, reflecting nucleosome spacing extending from transcription factor CTCF-binding sites, whereas the WPSs for the shorter DNA fragments show a peak centered on the CTCF-binding sites (green hexagon; Fig. 3b). Analysis of the nine individual datasets showed that nucleosome positioning arrays were highly reproducible between datasets, with variations within the range expected for plasma samples collected on different days (ρ > 0.93 for all pairwise combinations; Supplementary Fig. S10). Analysis of dinucleotide frequencies over 240-bp genomic windows centered at the mid-point of 167-nt DNA fragments showed the expected enrichment of AT at the ends of the fragments and auxiliary AA/AT/TA/TT- and CC/CG/GC/GG-rich regions, corresponding to minor grooves facing inward to or outward from the histone surface, all signatures for nucleosomal-protected DNA fragments identified by MNase-seq (Fig. 3c)^6,38^.

**Figure 4:**
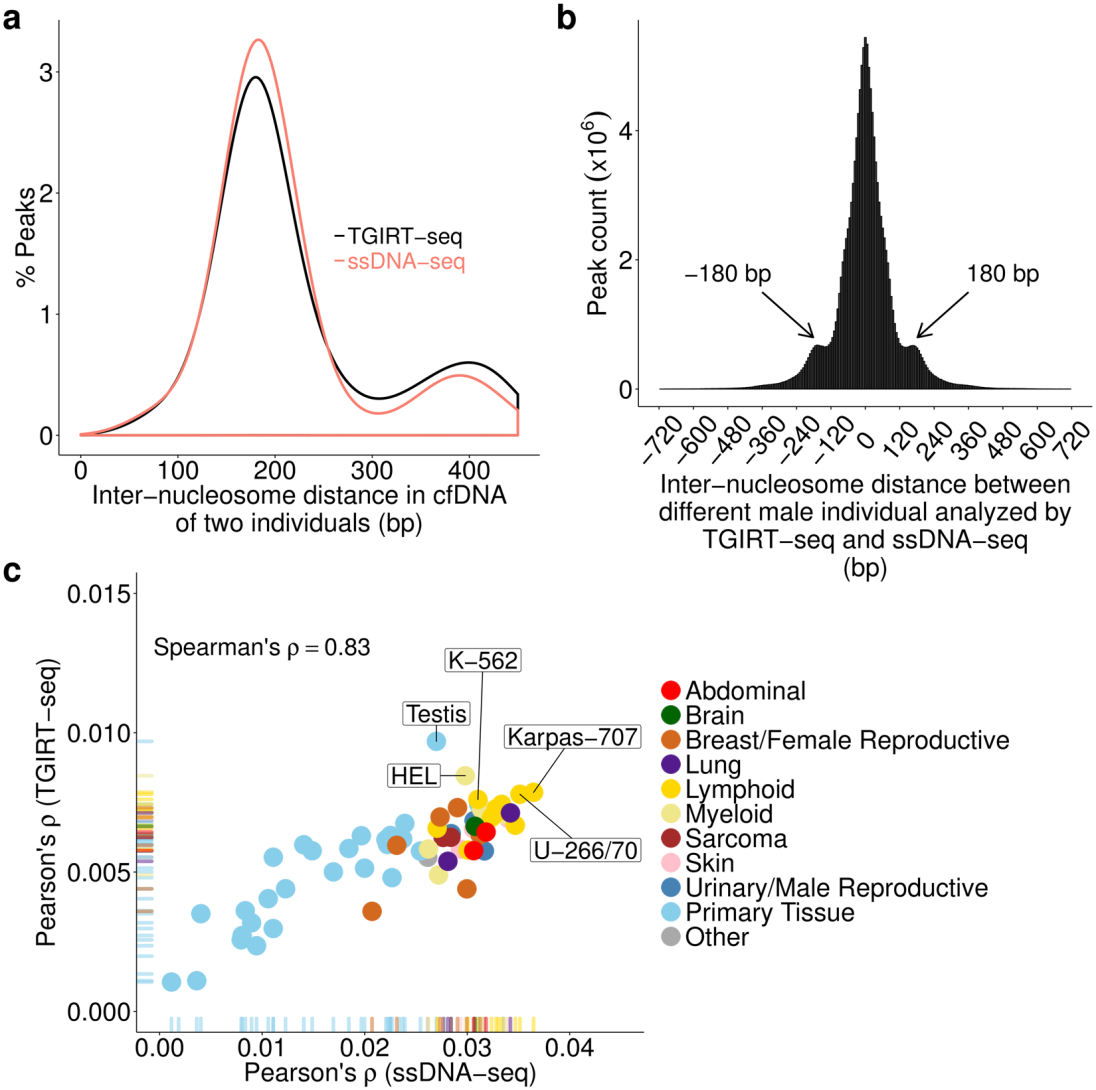
Comparisons of nucleosome landscapes of plasma DNA from two different healthy male individuals sequenced by TGIRT-seq and conventional ssDNA. (**a**) Plot of inter-nucleosome distances between nearest neighboring nucleosome centers in plasma DNA from two different healthy male individuals analyzed by TGIRT-seq (black line; combined datasets DP1-9) and conventional ssDNA-seq (pink line; SRR2130051). Both individuals show a peak distance of ~180 bp between nucleosome centers in their plasma DNA. (**b**) Histogram of distances (bin size of 6 bp) between the closest predicted nucleosome centers in plasma DNA from one healthy male individual analyzed by TGIRT-seq compared to those of the other healthy male individual analyzed by conventional ssDNA-seq (same datasets as panel (a)). Most predicted nucleosome centers having a nearest neighbor within ±1 kb are at the same position (distance = 0) or within one inter-nucleosome distance (subpeaks at 180 bp upstream or downstream) between the two individuals. (**c**) Tissues-of-origin of plasma cfDNA in the two healthy male individuals analyzed by TGIRT-seq and ssDNA-seq. Tissues-of-origin were calculated by correlating nucleosome occupancies in plasma DNA with gene expression levels for different cell lines and tissues in a published database^39^ as described by Snyder *et al*.^6^. Nucleosome occupancies within 10 kb downstream of a transcription start site in plasma DNA were calculated from the Fourier transform of the average intensity for 190–199 bp periodicity of window protection peaks and used to predict if a gene is expressed. The negative Pearson’s correlation coefficients (ρ) between spacing signal intensity and gene expression were used to rank tissue contributions. The overall Spearman’s rank-order correlation between the tissue rankings from TGIRT-seq and ssDNA datasets is 0.83. The most abundant sources of plasma DNA in both healthy male individuals are blood lymphoid and myeloid cells. The top 5 cell line and tissue matches for TGIRT-seq are labeled (HEL, erythroblast cell line; Karpas 707, human myeloma cell line; U266/70, B lymphocyte; K562, erythroleukemia cell line).

A comparison of inter-nucleosome distances showed that most of the predicted nucleosome centers in the TGIRT-seq dataset for a healthy male individual were within ~180 bp of each other, similar to findings obtained previously for a different healthy male individual by conventional ssDNA-seq (177 and 179 bp for TGIRT-seq﻿ and ssDNA-seq, respectively;﻿ Fig. 4a). To compare the predicted nucleosome-binding sites between the two healthy male individuals sequenced by TGIRT-seq and ssDNA-seq, we overlapped the datasets and computed distances between the TGIRT-seq peak-centers and their closest peak-centers from the ssDNA-seq datasets (Fig. 4b). This comparison showed that most predicted nucleosomal centers in the two healthy individuals are at the same site (peak at distance 0) or within one inter-nucleosome distance (sub-peaks at 180 bp upstream or downstream) with only a small proportion (4%) at longer distances, reflecting individual differences.

Finally, we compared the tissues-of-origin of plasma cfDNA in the two healthy male individuals by calculating nucleosome occupancies based on WPSs downstream of all annotated protein-coding gene transcription start sites for long cfDNA fragments (120–180 nt) and comparing them with gene expression levels for 76 human cell lines and primary tissues as described^6,39^. We found that the contribution rank of different tissues between the two individuals has a Spearman’s correlation of 0.83, with TGIRT-seq showing similar enrichment of nucleosomes derived from blood lymphoid or myeloid cells, but with a somewhat higher contribution from testis for the individual sequenced by TGIRT-seq (Fig. 4c). We conclude that TGIRT ssDNA-seq can be used to analyze nucleosome positioning, transcription factor-binding sites, and tissues-of-origin of cfDNA in human plasma similarly to conventional ssDNA-seq.

### Use of TGIRT-seq for bisulfite sequencing of plasma DNA

Another recent study showed that in addition to nucleosome positioning, the identification of DNA methylation sites in CpG islands by bisulfite sequencing can also be used to identify tissues-of-origin of cfDNAs^7^. Bisulfite treatment distinguishes between unmodified Cs and modified 5mC or 5hmC by converting the former but not the latter to Us^40^. However, bisulfite treatment also damages DNA making it difficult to generate high-quality DNA-seq libraries from such DNA by conventional methods^6,40^.

To test the suitability of TGIRT-seq for bisulfite sequencing, 5 ng of bisulfite-treated plasma DNA from the same healthy male individual was used directly to construct TGIRT-seq libraries following the workflow of Fig. 1. As noted previously, the construction of DNA-seq libraries from bisulfite-treated DNA prior to adapter addition is advantageous when working with small amounts of starting material as in the case for cfDNAs^41^. Comparison of the fragment length distributions in the datasets for bisulfite-treated versus untreated plasma DNA from the same individual shows the expected decrease in fragment size following bisulfite treatment, which is no impediment to TGIRT-template switching (see Supplementary Fig. S9). Mapping statistics for three TGIRT-seq libraries (BPD1-3) constructed from bisulfite-treated plasma DNA are summarized in Supplementary Table S4.

**Figure 5:**
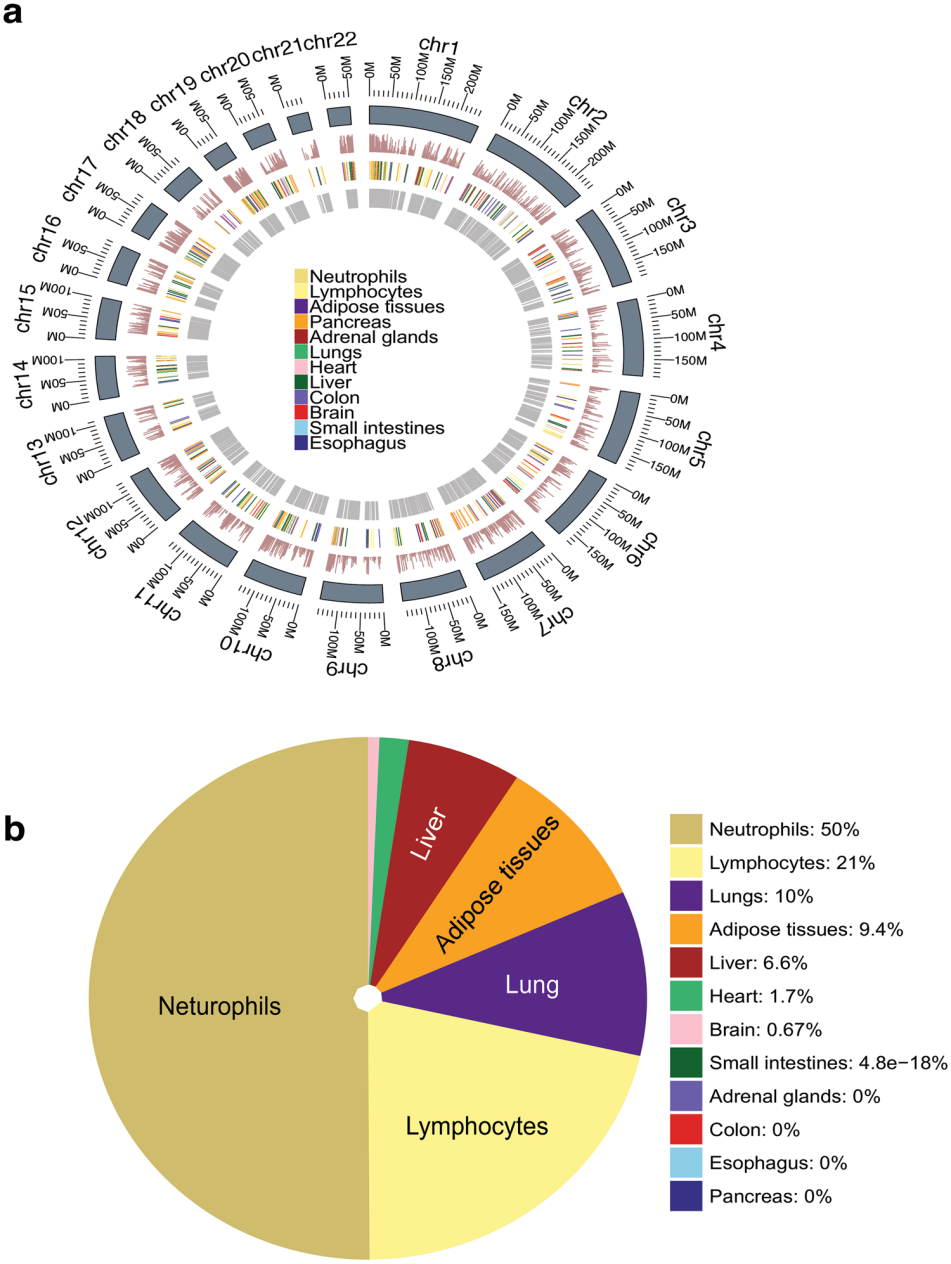
Bisulfite sequencing of plasma cfDNA. Three separate TGIRT-seq libraries were each constructed from ~5 ng of bisulfite-treated plasma DNA from a healthy male individual, and sequenced to obtain TGIRT-seq datasets BPD1-3 (see Supplementary Table S4). The datasets were combined and analyzed to identify DNA methylation sites, as described in Methods. (**a**) Annotation of DNA methylation sites in the human genome and determination of DNA methylation densities by TGIRT-seq of bisulfite-treated plasma cfDNA. Tracks from the inner to the outer circle represent: (1) annotations of Type II biomarkers that are highly variable in methylation density across tissues; (2) annotations of Type I biomarkers that are highly tissue specific (color-coded); (3) bar graphs of methylation densities within the annotated regions based on TGIRT-seq of bisulfite-treated plasma cfDNA; (4) genome coordinates. (**b**) Tissues-of-origin of plasma cfDNA from a healthy male individual determined by TGIRT-seq of bisulfite-treated DNA. The pie chart shows the percent contributions of different tissues in combined datasets PB1-3 determined by quadratic programming^7^. Neutrophils are derived from myeloid precursors, and lymphocytes are a combination of T-cells and B-cells. Comparison of tissues-of-origin of plasma cfDNA determined as in panel (**b**) from the three independent datasets BPD1-3 are shown in Supplementary Fig. 11. Pearson’s correlation coefficients were ≥0.96 for each pairwise combination of the three individual datasets.

To identify tissue of origins of this cfDNA, we combined the three TGIRT-seq datasets to obtain maximum information content and computed the average methylation densities in CpG islands from different annotated biomarker regions in the genome after error-correcting the sequencing reads using UMI clusters and removing any SNP positions (Fig. 5a)^7^. The published methylation biomarkers are 500-bp regions that can either be highly tissue specific (Type I biomarkers) or variably methylated across tissues (Type II biomarkers)^7^. We found that methylation densities of neutrophils and lymphocytes-specific biomarkers are high in plasma cfDNA from the healthy male individual, as expected (Fig. 5a).

To assess the contribution of different tissues to the cfDNA fragments, we applied a published tissue deconvolution method involving quadratic programming^7^. In agreement with tissues-of-origin identified by nucleosome positioning, this analysis showed that neutrophils (myeloid lineage) and lymphocytes (lymphoid lineage) are the major contributors to this plasma cfDNA identified by bisulfite sequencing (71% in total). This tissues-of-origin analysis was highly reproducible for each of the three datasets obtained for bisulfite-treated cfDNA extracted from three separate plasma samples (ρ ≥ 0.96 for each pairwise combination; see Supplementary Fig. S11). Together, these findings indicate that TGIRT-seq of bisulfite-treated DNA can be used to identify DNA methylation sites and identify tissues-of-origin of cfDNA comparably to conventional methods.

## Discussion

In this study, we show that a TGIRT enzyme can be used for ssDNA-seq and developed a streamlined TGIRT-based ssDNA-seq protocol that takes advantage of the novel template-switching activity of TGIRTs for DNA-seq library construction. Initial tests of the method for the sequencing of *E. coli* genomic DNA showed that the TGIRT enzyme copies DNA templates surprisingly well for an RT, with manageable errors rates and uniformity of coverage comparable to Nextera-XT. We then showed that TGIRT-seq of human plasma DNA enabled analysis of nucleosome positioning, transcription factor-binding sites, DNA methylation sites, and tissues-of-origin essentially identically to more cumbersome and/or expensive conventional ssDNA methods.

The TGIRT-seq of K12 *E. coli* genomic DNA showed the TGIRT-III enzyme gives an overall uniformity of coverage similar to Nextera-XT, including for regions having a wide-range of GC contents, as judged by Gini coefficients (0.26 and 0.28, respectively; Fig. 2c). At the extremes, TGIRT-seq gives better coverage of AT-rich regions than Nextera-XT, while over-representing regions of very high GC content (>60%). The latter was traced to end biases in the ssDNA-ligation step that adds a second DNA-seq adapter to the initial TGIRT DNA product and likely reflects inherent biases of this ligase enzyme^32^. As shown previously for RNA-seq, TGIRT template-switching introduces minimal sequence bias at the 3′ end of the DNA fragment^25,26^. Although the ligation bias at the 5′ end of the DNA fragment is not considered a major limitation, it might be mitigated by using a less biased ligase, adding polyethylene glycol to the ligation reaction, or by double-stranded adapter ligation^31,42^.

In this study, we used a 13-nt UMI at the 5′ end of the ligated R1R adapter for correction of PCR and sequencing errors. The length of the UMI was longer than needed at the current sequencing depth, but has the additional advantage of decreasing ligation bias compared to R1R adapters with a fixed sequence at their 5′ end (see Supplementary Fig. S5). Shorter UMIs as well as split UMIs in both the R1R and R2R adapters should also be feasible but remain to be tested. UMI resampling was found to be minimal (*i.e*., the number of identical fragments with different UMIs was not statistically distinguishable from that expected for DNA fragmentation and ligation biases in simulations; see Supplementary Fig. S8), possibly reflecting that TGIRT enzymes bind very tightly to and dissociate slowly from the completed DNA products.

After correction for errors introduced during PCR and sequencing by UMI clustering, base substitution rates for the TGIRT enzyme copying *E. coli* genomic DNA were similar to those for the Nextera-XT datasets, which are likely limited by the substitution rate of Illumina sequencing^35,36^. Indel rates for TGIRT-seq of DNA were consistently higher than that for Nextera-XT, but this was found to be due almost entirely to greater tendency for slippage at mononucleotide runs and could be dealt with bioinformatically. Multiple template-switches, a potential artifact that would decrease the fidelity of TGIRT-seq, were found to be minimal under the reaction conditions used in this study both for laboratory fragmented *E. col*i genomic DNA and for shorter plasma DNA fragments (see Supplementary Tables S2 and S3).

TGIRT-seq of plasma DNA from a healthy male individual enabled analysis of nucleosome positioning, transcription-factor binding sites, and DNA methylation sites essentially identically to conventional methods in recently published studies^6,7^. Moreover, analysis of nucleosome positioning and DNA methylation sites by TGIRT-seq identified tissue contribution to plasma DNA matching those of other healthy individuals analyzed by conventional methods in previous work^6,7^. TGIRT template-switching may be particularly advantageous for creating high quality DNA-seq libraries directly from damaged bisulfite-treated DNA.

The ability of TGIRT-III, a derivative of the thermostable GsI-IIC group II intron RT, to copy DNA templates relatively efficiently is surprising in light of previous studies of the contribution of the group II intron RT to retrohoming of the mesophilic Ll.LtrB group II intron, which has been used extensively as a model system for studying group II intron mobility. These studies showed that although the Ll.LtrB intron uses its encoded RT in the initial steps of retrohoming to synthesize a full-length cDNA copy of the intron RNA integrated into the genome, unlike retroviruses it relies on host DNA polymerases for second-strand DNA synthesis^43,44^. Initial biochemical experiments indicated that the Ll.LtrB intron RT had low DNA-dependent DNA polymerase activity^43^, but later studies showed this reflects primarily inefficient initiation from a DNA primer annealed to a DNA template compared to an otherwise identical DNA primer/RNA template combination^45^. This difference likely reflects that group II intron RTs prefer initiating DNA synthesis from a predominantly A-form rather than a B-form duplex and also holds true for template switching. In the configuration used for TGIRT-seq, the enzyme initiates DNA synthesis by template-switching from a synthetic RNA/DNA heteroduplex and template switches with similar efficiency to RNA or DNA templates^24^.

Based on our results, we anticipate that TGIRT-seq could be used in place of conventional ssDNA-seq methods for diagnostic applications involving analysis of cfDNAs in bodily fluids, as well as for the analysis of other highly fragmented DNA samples, such as ancient DNA and DNA from FFPE tumor slices. More accurate mutation detection might be achieved for TGIRT-seq by higher sequencing depth or by using more complex bioinformatic pipelines using both strands (two read clusters) of the same dsDNA fragments^46^. As noted previously, however, ssDNA-seq methods are not expected to replace established targeted DNA-seq methods, such as Safe-SeqS and CAPP-seq, for mutation detection^6,13,47^. Rather their unique advantages are for the sequencing of highly fragmented DNA samples, separately analyzing individual DNA strands, and characterizing epigenetic features, such as nucleosome positioning, transcription factor-binding sites, and DNA methylation sites. The latter enables determination of the tissues-of-origin of cfDNAs in a variety of pathological conditions without relying on differences in genomic sequence^6,7^. TGIRT-seq offers a new efficient, streamlined, and relatively low cost ssDNA-seq method that should be useful for a variety of applications.

## Methods

### Ethics statement

De-identified excess (discarded) plasma was used from a prior study^25^, which had been declared to not meet the requirements for human subjects research as defined by the Common Rule (45 CFR 46) or FDA Regulations (21 CFR 50 & 56) by the University of Texas Office of Research Support.

### Preparation of *E. coli* DNA

*E. coli* K12 (MG1655) genomic DNA was obtained from the American Type Culture Collection (ATCC 700926). For each DNA-seq library, the DNA (500 ng in 130 μl of 10 mM Tris-HCl, pH 7.5, 1 mM EDTA (TE)) was fragmented to a target size of 400 bp with a Covaris sonicator (S220, Woburn, MA) using a 10% duty cycle, intensity at 5, and 200 cycles per burst. After sequential size-selection with 0.6X and 1X Agencourt AMPure XP beads (Beckman Coulter) to remove larger and smaller DNA fragments, respectively, the remaining DNA fragments used for library preparation had an average length of ~250 bp.

### TGIRT-seq of *E. coli* genomic DNA

Fragmented *E. coli* genomic DNA (8–50 ng) was dephosphorylated with FastAP (1 unit; Thermo Fisher) in 13.2 μl of a solution containing 20 mM Tris-HCl pH 7.5, 5 mM MgCl_2_, and 7.5 mM dithiothreitol (DTT) for 20 min at 37 °C and denatured by heating at 95 °C for 5 min. The DNA was then used as substrate for TGIRT template-switching DNA synthesis from an initial template-primer substrate consisting of a 34-nt RNA oligonucleotide (R2 RNA), which contains an Illumina Read 2 (R2) primer-binding site and a 3′-blocking group (C3 Spacer, 3′SpC3; IDT), annealed to a complementary 35-nt DNA primer (R2R DNA), which leaves an equimolar mixture of A, C, G, or T single-nucleotide 3′ overhangs (see Supplementary Table S1). Reactions were done with 5–50 ng DNA substrate, 100 nM annealed template-primer substrate, and TGIRT-III enzyme (400 units, 1 mM; InGex, LLC; St. Louis) in 20 μl of reaction medium containing 420 mM NaCl, 5 mM MgCl_2_, 20 mM Tris-HCl, pH 7.5, 5 mM DTT and 1 mM dNTPs (an equimolar mix of 1 mM dATP, dCTP, dGTP, and dTTP). Prior to the reactions, the template-primer substrate was pre-incubated with TGIRT-III enzyme for 30 min at room temperature in 6 μl of reaction mixture containing 1.4 M NaCl, 5.8 mM MgCl_2_, 23.3 mM Tris HCl pH 7.5 and then mixed with the 13.2-μl dephosphorylation reaction mixture containing the DNA substrate (see above) before initiating template-switching and DNA synthesis by adding dNTPs. Reactions were incubated at 60 °C for 40 min and terminated by adding 5 M NaOH to a final concentration of 0.25 M, incubating at 95 °C for 3 min, and then neutralizing with 5 M HCl. After cleaning up by using a Nucleospin Gel and PCR cleanup kit (Clontech), a 5′App/3′-CpSp blocked R1R adapter with a 13-nt UMI at its 5′ end (denoted R1R-UMI) was ligated to the 3′ end of the cDNA by using Thermostable 5′ AppDNA/RNA Ligase as specified by manufacturer’s protocol (New England Biolabs). The ligated products were cleaned up by Nucleospin Gel and PCR cleanup kit (Clontech) and PCR amplified by using a KAPA Library Amplification Kit (KAPA) with 500 nM each of Illumina multiplex and barcode primers, with the 5′ primer adding a P5 capture site and the 3′ primer adding a sequencing barcode and P7 capture site (see Supplementary Table S1). PCR was done with initial denaturation at 98 °C for 30 sec followed by 10–12 cycles of 98 °C for 45 sec, 60 °C for 15 sec, and 72 °C extension for 30 sec, with a final extension of 72 °C for 5 min. The PCR products were size-selected with 1X Agencourt AMPure XP beads (Beckman Coulter) to remove adapter dimers, and then sequenced on a NextSeq 500 or HiSeq 4000 (Illumina) to obtain 2 × 75 nt or 2 × 150 nt paired-end reads (see Supplementary Table S2). Additional libraries were constructed with other variations of the R1R adapter (an R1R adapter without a UMI or an R1R with the 13-nt UMI preceded by a 5-bp fixed sequence (R1R-UMI-CGATG; see Supplementary Table S1).

### *E. coli* genomic DNA-seq bioinformatic analysis

For read mapping of TGIRT-seq data, the first 13 bases (UMI sequence) of Reads 1 with an average Phred score >20 were detached from the sequence and attached to the read ID. For experiments using an R1R adapter in which the 13-nt UMI is preceded by a short fixed sequence, up to 2 mismatches in the fixed sequence were permitted for retention of the read pair. For comparisons, Nextera-XT DNA-seq datasets obtained for the same *E. coli* genomic DNA were downloaded from the Illumina Basespace (samples 10, 50 and 60; Illumina Basespace project 21071065: NextSeq 500 V2: Nextera-XT 384-plex *E. coli*)^29^ and clipped to 75-nt prior to data processing. Both the TGIRT-seq and Nextera-XT paired-end reads were adapter trimmed with Trimmomatic v0.36 using the following parameters: ILLUMINACLIP:adaptors.fa:2:10:10:1:true LEADING:10 TRAILING:10 SLIDINGWINDOW:4:8 MINLEN:18 AVGQUAL:15, using a customized adaptors.fa for TGIRT-seq data (https://gist.github.com/wckdouglas/a4ce119ebbcddb543b6c487d587fee89) and the NexteraPE-PE.fa adapter file supplied by the Trimmomatic package for Nextera-XT data^48^. A FASTA file for *E. coli* K12 genome DNA (NC_000913.3) was downloaded from NCBI and indexed. Trimmed reads were mapped to the NC_00913.3 by BWA v0.7.15 using default parameters, and alignments were sorted by coordinates with SAMtools v1.3^49,50^. Alignments were filtered to retain proper pairs of concordant reads (flag: 83, 99, 147 and 163), and PCR and optical duplicates were removed by *MarkDuplicates* from Picard tools v2.5 (Broad Institute). For error correction, UMIs were used for deduplication and consensus base calling using a customized program employing a Bayesian algorithm described previously^51^. All cluster families regardless of size were retained. Whole genome sequencing metrics were analyzed with 10 subsampled datasets, each consisting of 1 million reads (~16X coverage of the *E. coli* genome) randomly sampled from technical replicates by BEDtools v2.26^52^, except for determination of GC biases, where full datasets were analyzed. GC bias, coverage bias and base error metrics were extracted by *CollectAlignmentSummaryMetrics, CollectGcBiasMetrics* and *CollectRawWgsMetrics* with Picard tools v2.5 (Broad Institute). Gini coefficients are computed by *ineq* package from R.

Base substitution rates, indel rates, and nucleotide composition biases were extracted by customized scripts using *Pysam* package^50^. Simulated datasets for the *E. coli* K12 genome were created with customized scripts using *pyFaidx* and *SciPy* packages following a previously described algorithm^6,53,54^. All plots were done with *ggplot2* or *seaborn* and *maplotlib*^55–57^.

### Preparation of human plasma DNA

cfDNA was isolated from plasma of a healthy male individual obtained from the Genome Sequencing and Analysis Facility at the University of Texas at Austin. To prepare plasma, fresh blood was collected in 10-ml K^+^/EDTA venous blood collection tubes, mixed with an equal volume of phosphate-buffered saline without calcium or magnesium (PBS −/−; Thermo Fisher Scientific), gently layered over 15-ml Ficoll-Paque PLUS (GE Healthcare) in a 50-ml conical tube, and centrifuged at 400 × g for 35 min at room temperature. After centrifugation, plasma (top layer) was transferred into a clean tube and divided into 1-ml portions, which were stored at −80 °C.

To extract DNA, 400 μl of plasma was incubated with RNase I (Qiagen; 800 μg) as suggested by the manufacturer’s protocol and then processed by using a Qiagen DSP DNA Blood Mini Kit (Qiagen). The products were mixed and concentrated with an Oligo Clean and Concentrator kit (Zymo) and eluted in 10 μl of water per 1 ml plasma. The extracted DNA was analyzed by using a Bioanalyzer High Sensitivity DNA Analysis Kits (Agilent) and consistently contained 2–3 ng of predominantly 150–170 bp fragments per 1-ml plasma (see Supplementary Fig. S9).

### TGIRT-seq of human plasma DNA

TGIRT-seq of human plasma DNA was done as described above for *E. coli* genomic DNA, except that that the input was 2–2.5 ng DNA, the reaction time was decreased to 20 min, and the DNA products were cleaned up with a MinElute Cleanup Kit (Qiagen) prior to PCR. For PCR, annealing was for 10 sec at 60 °C and extension was for 10 sec at 65 °C to accommodate the shorter DNA products. The PCR products were cleaned up with 1.3X Agencourt AMPure XP beads (Beckman Coulter) to remove adapter dimers.

### Plasma DNA-seq bioinformatic analysis

For TGIRT-seq data, the first 13 bases (UMI sequence) of Reads 1 with an average Phred score >20 were detached from the sequence and attached to the read ID. For experiments using an R1R adapter in which the 13-nt UMI is preceded by a short fixed sequence, up to 2 mismatches in the fixed sequence were permitted for retention of the read pair. For comparisons, published paired-end plasma ssDNA-seq datasets for a healthy male and a healthy female individual (SRR2130051 and SRR2130052) were downloaded from the Sequence Read Archive (SRA) using command: *fastq-dump -split-3 -gzip* from SRA-toolkit v2.8 (NCBI). The TGIRT-seq paired-end reads were adapter trimmed with the parameters described above for *E. coli* genomic DNA, and trimming of the published data was done with TruSeq2-PE.fa from the Trimmomatic package. Trimmed paired-end reads were aligned to a human genome reference sequence supplemented by additional of rRNA sequences (GRCh38 version 76, gi|555853|gb|U13369.1|HSU13369 and gi|23898|emb|X12811.1|), by using the MEM algorithm in BWA v0.7.15^49^. Alignments with flags 4, 8, 256, 512, 1024 and 2048 were filtered with SAMtools v1.3, and all concordant read pairs were converted into genome coordinates using BEDtools *bamtobed* with arguments *-mate1 -bedpe*^50,52^. PCR deduplication was done by using custom scripts to identify reads with the same UMI and the same coordinates for both fragment ends, while allowing up to 1 mismatch in the UMI for TGIRT-seq data; UNIX *sort* and *uniq* commands for published ssDNA-seq data. Window protection scores and nucleosome peak calling were done with previously described algorithms^6^ with customized python scripts utilizing *NumPy, SciPy, pyBigWig*, *Pysam* and *Pybedtools* API^50,53,58,59^. Fourier transforms of nucleosome positions were done by using a previously described algorithm implemented using *SciPy, statsmodel* and *NumPy* in python. All plots were done under R with *ggplot2*^56^.

### Bisulfite sequencing

Plasma cfDNA (20 ng), which had been isolated as described above, was concentrated to 20 μl with an Oligo Clean and Concentrator kit (Zymo). The concentrated DNA was treated with EZ DNA Methylation-Lightning kit (Zymo) following the manufacturer’s protocol and eluted in 10 μl of elution buffer supplied by the kit. TGIRT-seq libraries were prepared using the workflow shown in Fig. 1 as described above for *E. coli* genomic DNA, starting with 5 ng of bisulfite-treated plasma DNA. The libraries were sequenced on an Illumina NextSeq 500 to obtain ~ 200 million 2 × 75 nt reads.

For read mapping, the first 13 bases (UMI sequence) of Reads 1 with an average Phred score >20 were detached from the sequence and attached to the read ID. The reads were then mapped to the human genome reference sequence (hg19) using bwa-meth v0.2.0^60^. Read pairs with same UMI and mapping coordinates were clustered and consensus base were called from the family members in the read cluster using a customized program implementing a previously described algorithm^51^.

To identify methylated C residues, the consensus read pairs were adapter- and quality-trimmed using the parameters described above for *E. coli* genomic DNA except setting MINLEN as 15, and the trimmed reads were re-mapped to human genome reference (hg19) using bwa-meth v0.2.0^60^. Methylation densities at CpG sites were extracted by MethylDackel v0.2.1 using options -q 1 -p 25 -d 2 -D 2000 -fraction (https://github.com/dpryan79/MethylDackel), and CpG sites overlapping hg19 SNPs were filtered out. Methylation biomarker annotations were downloaded from Supplementary Dataset S01 of Sun *et al*.^7^, and Type I and II biomarkers were merged into a single annotation table. TGIRT-seq average methylation density for these regions were calculated using a combination of BEDtools and datamash^52^. Tissue distributions were computed using *quadprog* and plotting were done by *ggplot2* and *ggbio* in R^56,61^.

### Statistical analysis

A two-sample two-tailed Wilcoxon-Mann-Whitney test and a two-sample two-tailed F-test were used for statistical tests comparing TGIRT-seq and Nextera-XT for base coverage and GC coverage. Summary statistics showed means and standard deviations for each group. A one-way Chi-square test was used for template reutilization.

### Code availability

All scripts used for data processing is deposited on GitHub: https://github.com/wckdouglas/tgirt-dna-seq.

### Accession numbers

The sequencing data sets in this manuscript have been deposited in the National Center for Biotechnology Information Sequence Read Archive (http://www.ncbi.nlm.nih.gov/sra) under accession number SRP107051.

## Electronic supplementary material

Supplementary Information
