# Author Correction: Facile single-stranded DNA sequencing of human plasma DNA via thermostable group II intron reverse transcriptase template switching

**DOI:** 10.1038/s41598-018-21665-7

**Published:** 2018-03-01

**Authors:** Douglas C. Wu, Alan M. Lambowitz

**Affiliations:** 1 Institute for Cellular and Molecular Biology, University of Texas at Austin, Austin, Texas 78712 USA; 2 Department of Molecular Biosciences, University of Texas at Austin, Austin, Texas 78712 USA

Correction to: *Scientific Reports* 10.1038/s41598-017-09064-w, published online 21 August 2017

This Article contains a typographical error.

In the Methods section under subheading ‘TGIRT-seq of E. coli genomic DNA’,

“(400 units, 1 mM; InGex, LLC; St. Louis)”

should read:

“(400 units, 1 µM; InGex, LLC; St. Louis)”

