# Supplementary material for: Facile single-stranded DNA sequencing of human plasma DNA via thermostable group II intron reverse transcriptase template switching
Source: Sci Rep. 2017 Aug 21;7:8421. doi: 10.1038/s41598-017-09064-w (PMC5566474; doi:10.1038/s41598-017-09064-w)
Supplement: Supplementary file 1 — Supplementary Information [file 41598_2017_9064_MOESM1_ESM.pdf]

Facile single-stranded DNA sequencing of human plasma DNA via thermostable group II intron reverse transcriptase template switching

Douglas C. Wu[1,2] and Alan M. Lambowitz[1,2,*]

[1]Institute for Cellular and Molecular Biology
[2]Department of Molecular Biosciences
University of Texas at Austin
Austin, Texas, 78712, USA

Key Words: Bisulfite sequencing, diagnostics, DNA methylation, nucleosome positioning

*Corresponding author

Supplementary Table 1: Oligonucleotides used in the experiments.

Name	Sequence
R2 RNA	5'- AGA UCG GAA GAG CAC ACG UCU GAA CUC CAG UCA C -3' SpC3
R2R DNA	5'- GTG ACT GGA GTT CAG ACG TGT GCT CTT CCG ATC TN -3'
R1R	5'- App GA TCG TCG GAC TGT AGA ACT CTG AAC GTG TAG -3' SpC3
R1R-UMI	5'- App NNN NNN NNN NNN NGA TCG TCG GAC TGT AGA ACT CTG AAC GTG TAG -3' SpC3
R1R-UMI+CGATG	5'- App CGA TGN NNN NNN NNN NNN GAT CGT CGG ACT GTA GAA CTC TGA ACG TGT AG -3' SpC3
Multiplex PCR primer (P5+Read 1)	5'- AAT GAT ACG GCG ACC ACC GAG ATC TAC ACG TTC AGA GTT CTA CAG TCC GAC GAT C -3'
Barcode primer (P7 + barcode + read2)	5'- CAA GCA GAA GAC GGC ATA CGA GAT NNN NNN GTG ACT GGA GTT CAG ACG TGT GCT CTT CCG ATC T -3'
50-nt test oligo	5'- ATA AGC TTT AAT ACG ACT CAC TAT AGG GCA CTT TTT CCA TCC CTT TTC GC -3'
78-nt test oligo	5'- CTA TAC ATC AGT GTG TGA CAT CTG TGA TGA GCA CGT GTG ACT GAT AGC TGA TGA TCT GCG AGA TCN NNN NNN NNN NNN -3'
90-nt test oligo	5'- GTG ACT GGA GTT CAG ACG TGT GCT CTT CCG ATC TNN NNN NNN NNN NNN NTA CGC TCT TTC TCC GCG AAT GCG GCG AGC GAG CTG GAT GTC -3'
R1 qPCR primer	5'- CTA CAC GTT CAG AGT TCT ACA GTC CGA CGA TC - 3'
R2R qPCR primer	5'- GTG ACT GGA GTT CAG ACG TGT GCT CTT CCG ATC T -3'

The order of primers in the table corresponds to the order of their use in the text. Illumina Read 2 (R2) RNA and Read 2 reverse (R2R) DNA are the annealed template-primer substrate used to initiate copying of a single-stranded DNA by template-switching. Illumina Read 1 reverse (R1R) is the oligonucleotide ligated to the 3' end of the DNA product. Three versions of the R1R DNA oligonucleotide without (R1R) or with a 13-nt UMI directly at its 5' end (R1R-UMI) or with the UMI preceded by a short fixed sequence (R1R-UMI+CGATG) were used in the work. Multiplex PCR primer, and Barcode Primer add Illumina P5 and P7 adapter priming sites and sample barcodes for Illumina sequencing. Read 1 qPCR (R1) and Read 2 reverse (R2R) qPCR primers were used for qPCR assay of R1R DNA ligation efficiency. All oligonucleotides were obtained from IDT (San Jose, CA). N indicates machine mixed random nucleotides. 3'SpC3 is a three-carbon blocker that prevents template switching or ligation to the 3' end of an oligonucleotide. 5'App indicates a 5' adenylated oligos.

Supplementary Table 2: *E. coli* K12 MG1655 genomic DNA-seq statistics.

	TGIRT-seq							Nextera-XT		
**Sample ID**	EG1	EG2	EG3	EG4	EG5	EG6	EG7	NX10	NX50	NX60
**Fragmentation**	Covaris	Covaris	Covaris	Covaris	Covaris	Fragmentase	Fragmentase	Transposon	Transposon	Transposon
**R1R primer**	R1R-UMI	R1R-UMI	R1R-UMI	R1R-UMI+CGATG	R1R-UMI+CGATG	R1R-UMI+CGATG	R1R-UMI+CGATG	N/A	N/A	N/A
**Sequencer**	NextSeq 500	NextSeq 500	NextSeq 500	HiSeq 4000	HiSeq 4000	NextSeq 500	NextSeq 500	NextSeq 500	NextSeq 500	NextSeq 500
**Read Length**	2x75	2x75	2x75	2x150	2x150	2x75	2x75	2x150	2x150	2x150
**Raw pairs**	4,678,431	3,845,521	4,241,153	881,946	815,360	3,504,443	3,417,172	541,194	913,536	683,602
**UMI > Q20**	4,674,617 (99.9%)	3,842,227 (99.9%)	4,237,686 (99.9%)	702,106 (79.6%)	649,312 (79.6%)	3,272,736 (93.4%)	3,173,975 (92.9%)	541,194 (100.0%)	913,536 (100.0%)	683,602 (100.0%)
**Trimmed pairs**	4,350,888 (93.1%)	3,518,677 (91.6%)	3,992,699 (94.2%)	637,655 (90.8%)	583,815 (89.9%)	3,175,221 (97.0%)	3,068,202 (96.7%)	526,151 (97.2%)	894,275 (97.9%)	665,486 (97.3%)
**Mapped reads**	8,601,272 (98.8%)	6,991,356 (99.3%)	7,930,106 (99.3%)	1,269,785 (99.6%)	1,162,604 (99.6%)	6,316,656 (99.5%)	6,096,749 (99.4%)	1,050,078 (99.8%)	1,785,076 (99.8%)	1,328,502 (99.8%)
**Concordant reads**	8,584,856 (99.8%)	6,978,372 (99.8%)	7,915,326 (99.8%)	1,264,454 (99.6%)	1,157,776 (99.6%)	6,292,862 (99.6%)	6,066,548 (99.5%)	1,047,972 (99.8%)	1,779,862 (99.7%)	1,324,734 (99.7%)
**Duplicate reads**	4,327,645 (50.3%)	3,996,536 (57.2%)	4,396,793 (55.4%)	335,652 (26.4%)	311,860 (26.8%)	2,709,984 (42.9%)	3,294,452 (54.0%)	16,771 (1.6%)	32,919 (1.8%)	21,381 (1.6%)
**Chimeric reads**	145 (0.001%)	84 (0.001%)	107 (0.002%)	438 (0.03%)	486 (0.04%)	108 (0.001%)	217 (0.003%)	39 (0.003%)	388 (0.002%)	240 (0.001%)

Datasets EG1-7 were obtained by TGIRT-seq of *E. coli* K12 genomic DNA. Nextera-XT datasets NX10, 50 and 60 used for comparisons were downloaded from Illumina basespace project 21071065[29]. Raw pairs were calculated from raw FASTQ files after sample demultiplexing. UMI >Q20 indicates the number of read pairs having a mean UMI quality >20. Trimmed pairs indicate the number of UMI >Q20 read pairs after adapter trimming. Mapped reads are the number of reads mapped to the *E. coli* K12 genome. Concordant reads are reads having mapper assigned alignment bit flag 0x2. Duplicate reads are the number of reads determined to be duplicates by Picard tools (Broad institute). Chimeric reads are the number of reads with supplementary alignments.

Supplementary Table 3: Plasma DNA-seq statistics.

Method	TGIRT-seq									ssDNA-seq	
**Sample ID**	PD1	PD2	PD3	PD4	PD5	PD6	PD7	PD8	PD9	SRR2130051	SRR2130052
**R1R primer**	R1R-UMI+CGATG	R1R-UMI+CGATG	R1R-UMI+CGATG	R1R-UMI	R1R-UMI	R1R-UMI	R1R-UMI	R1R-UMI	R1R-UMI	N/A	N/A
**Raw pairs**	66,807,919	93,284,041	97,878,762	63,034,937	62,194,137	51,817,791	115,723,907	121,774,138	98,346,922	665,931,126	49,089,697
**UMI > Q20 pairs**	62,537,025 (93.6%)	87,689,383 (94.0%)	92,010,965 (94.0%)	62,979,109 (99.9%)	62,138,059 (99.9%)	51,770,492 (99.9%)	115,650,646 (99.9%)	121,685,193 (99.9%)	98,263,067 (99.9%)	N/A	N/A
**Trimmed pairs**	57,638,283 (92.2%)	62,479,326 (71.3%)	74,897,804 (81.4%)	54,555,929 (86.6%)	56,210,570 (90.5%)	45,815,664 (88.5%)	105,895,941 (91.6%)	100,864,791 (82.9%)	73,879,710 (75.2%)	643,716,574 (96.7%)	47,517,651 (96.8%)
**Mapped reads**	98,284,359 (85.3%)	106,341,051 (85.1%)	132,424,340 (88.4%)	90,059,587 (82.5%)	93,941,368 (83.6%)	76,018,611 (83.0%)	193,197,219 (91.2%)	190,191,312 (94.3%)	137,340,730 (92.9%)	1,225,396,311 (95.2%)	88,310,817 (92.9%)
**Concordant reads**	94,647,956 (96.3%)	103,162,434 (97.0%)	128,371,532 (96.9%)	88,179,312 (97.9%)	91,294,872 (97.2%)	73,930,294 (97.3%)	189,526,302 (98.1%)	185,350,478 (97.5%)	133,086,384 (96.9%)	1,203,514,980 (98.2%)	85,198,014 (96.5%)
**Chimeric reads**	69,511 (0.1%)	71,124 (0.1%)	77,847 (0.1%)	85,539 (0.1%)	97,125 (0.1%)	74,887 (0.1%)	141,328 (0.1%)	129,601 (0.1%)	92,119 (0.1%)	9,048 (0.0%)	0 (0.0%)

Datasets PD1-9 were obtained by TGIRT-seq of plasma DNA from a healthy male individual using R1R primers R1R-UMI+CGATG (PD1-3) or R1R-UMI (PD4-9) (see Table 1). The datasets obtained for the TGIRT-seq libraries were compared to those obtained for healthy male and female individuals by conventional ssDNA-seq (SRR2130051 and SRR2130052, respectively)[6]. Rows definitions are the same as in Supplementary Table S2. The somewhat lower proportion of trimmed pairs in the TGIRT-seq datasets likely reflect incomplete primer-dimer removal and might be improved by additional library clean up prior to sequencing.

Supplementary Table 4: TGIRT bisulfite-sequencing of human plasma DNA mapping statistics

Treatment	Sample ID	Raw pairs	UMI > Q20 pairs	Mapped reads	Concordant reads	Cluster pairs	Trimmed clusters pairs	Mapped clusters reads	Converted CpG	Converted Non-CpG
**Bisulfite-treated**	BPD1	179,954,644	162,424,950 (90.3%)	306,822,533 (94.5%)	306,016,372 (99.7%)	31,233,870 (20.4%)	27,879,583 (89.3%)	55,758,980 (100.0%)	28%	86.7%
**Bisulfite-treated**	BPD2	183,438,214	170,091,607 (92.7%)	331,708,403 (97.5%)	331,189,068 (99.8%)	43,473,967 (26.3%)	38,691,261 (89.0%)	77,093,093 (99.6%)	25.8%	87.1%
**Bisulfite-treated**	BPD3	211,218,882	197,947,224 (93.7%)	390,930,528 (98.7%)	390,279,660 (99.8%)	29,222,058 (15.0%)	24,513,729 (83.9%)	48,911,633 (99.8%)	26.1%	87.5%

The TGIRT-seq library was constructed using the workflow of Fig. 1 with the R1R-UMI DNA oligonucleotide. Cluster pairs are the number of error-corrected read pairs using UMI clusters from concordant read pairs. Trimmed clusters pairs indicates the number of cluster pairs that remained after adapter and quality trimmings. Nearly all cluster pairs could be mapped to the human genome. Converted CpG indicates the percentage of cytidines in CpG islands converted to thymidine after bisulfite treatment. Converted Non-CpG indicates the percentage of cytidines not followed by a guanosine that are converted to thymidine by bisulfite treatment. Other definitions are as in Supplementary Table 2.

Supplementary Figure S1

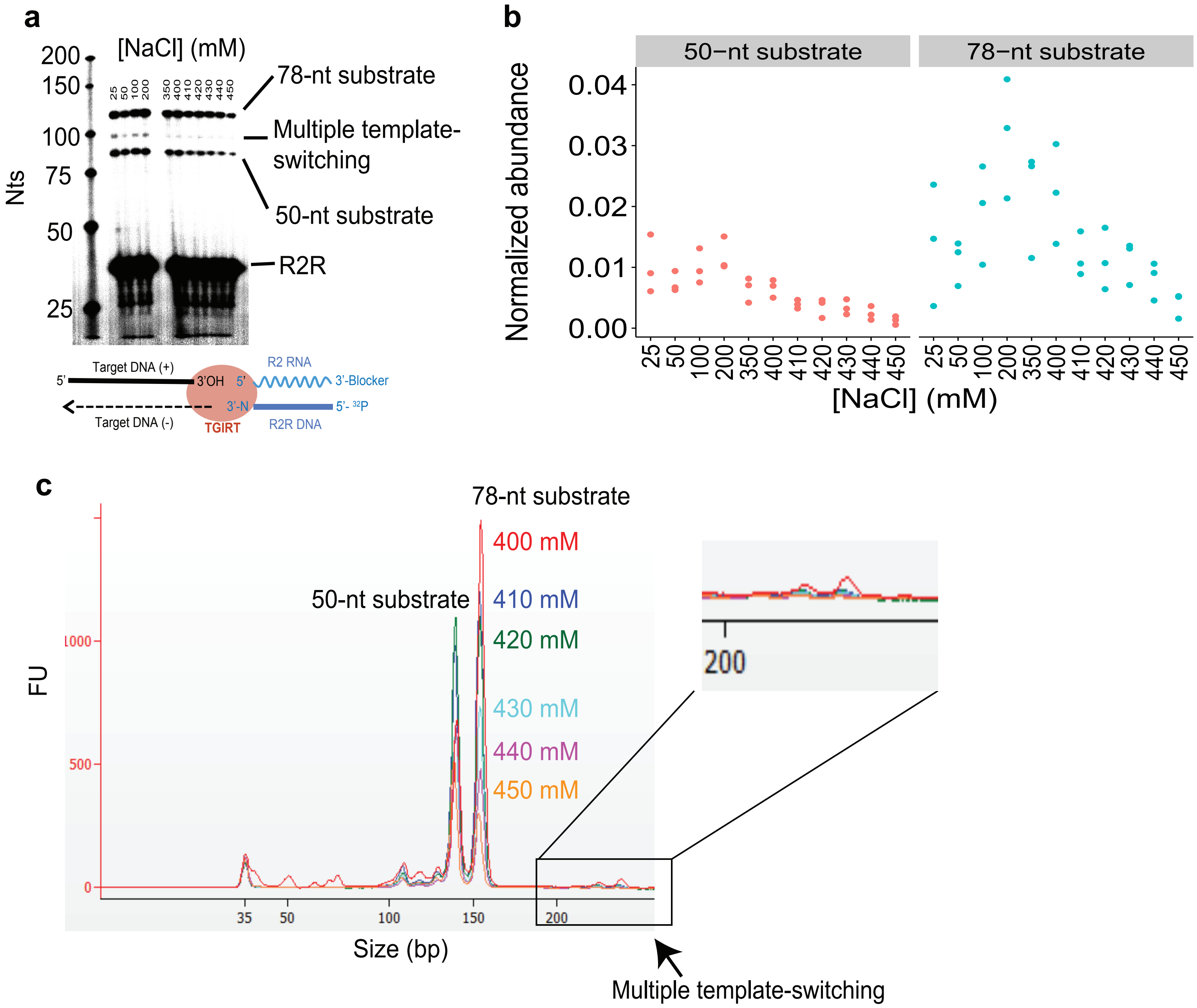

Supplementary Figure S1 / Effect of salt concentration on TGIRT-III template switching and DNA synthesis. TGIRT template-switching DNA synthesis reactions were done from an initial R2 RNA/R2R DNA template-primer substrate (1 µM) with 5' ^{32}P-labeled R2R DNA to a mixture of 50- and 78-nt test DNA oligonucleotides (0.25 µM each; Supplementary Table 1) for 15 min at 60 ^{o}C in reaction medium containing different NaCl concentrations. **(a)** Products of the template-switching DNA synthesis reactions were analyzed by electrophoresis in a denaturing 6% polyacrylamide gel against a low molecular weight DNA ladder (New England Biolabs). The figure shows an image of the gel obtained with a PhosphorImager (Typhoon 9600, GE Life Sciences), with a schematic of the reaction shown beneath the gel image. **(b)** Quantification of DNA products from three replicates of the experiment in panel (a). Products were quantified from their band intensities in PhospborImager scans by using ImageJ and expressed relative to that of the R2R DNA oligonucleotide in the same lane (3 replicates for each salt concentration). **(c)** Bioanalyzer trace of TGIRT-seq libraries prepared by template-switching to the 50- and 78-nt DNA oligonucleotide substrates at 400-450 nM NaCl. The expanded region of the bioanalyzer traces shows a substantial decrease in multiple template-switches at NaCl concentrations >400 mM. Abbreviation: FU, fluorescence units.

Supplementary Figure S2

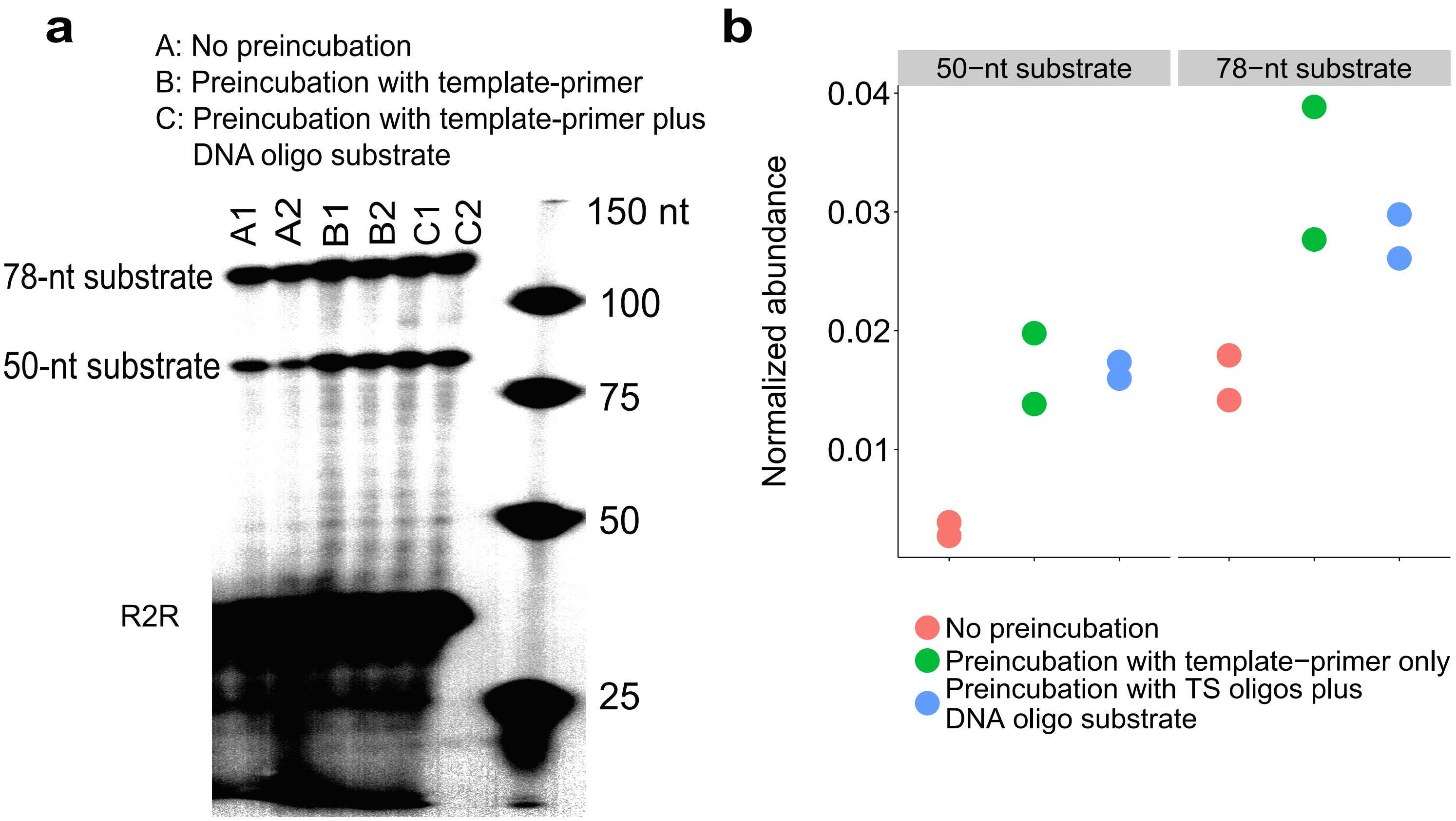

Supplementary Figure S2 / Effect of TGIRT-III pre-incubation with initial template/primer substrate on the efficiency of DNA synthesis. TGIRT template-switching DNA synthesis reactions with a mixture of 50- and 78-nt test DNA oligonucleotides substrates were done as described in Supplementary Fig. 1 in reaction medium containing 420 mM NaCl without or with pre-incubation of the TGIRT-III enzyme with the initial template-primer substrate by itself or together with the target DNA substrates for 30 min at room temperature. **(a)** Products of the template-switching DNA synthesis reactions analyzed by electrophoresis in a denaturing 6% polyacrylamide gel as in Supplementary Fig. 1a. **(b)** Quantitation of DNA products by PhosphorImager band intensity relative to labeled R2R DNA primer in the same lane. The results show that pre-incubation of the TGIRT enzyme with either the initial template/primer substrate or the initial template/primer substrate plus the DNA oligonucleotide substrates increases the efficiency of DNA synthesis.

Supplementary Figure S3

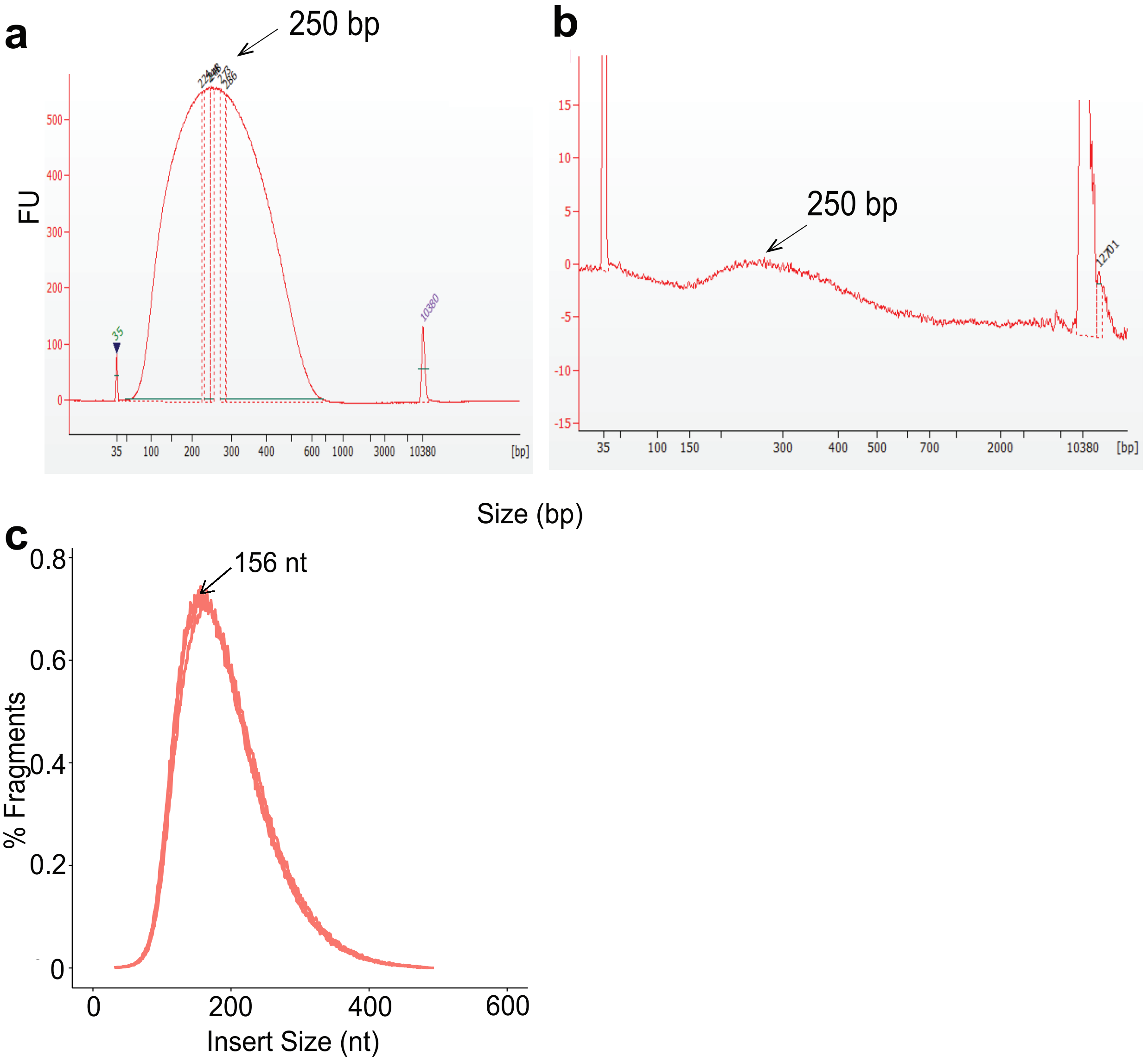

Supplementary Figure S3 / Comparison of *E. coli* DNA fragment size with fragment length distribution in a TGIRT-seq libary. (a) Bioanalyzer trace of *E. coli* K12 DNA fragmented by Covaris sonication followed by sequential size-selection with 0.6X and 1X Ampure beads to retain 100-600 bp fragments, as described in Methods. The trace was obtained with an Agilent Bioanalyzer 2100 using a High Sensitivity DNA chip. **(b)** Bioanalyzer trace of TIGRT-seq library prepared from the Covaris-fragmented *E. coli* K12 DNA in panel (a) analyzed using a High Sensitivity DNA chip. The broad size distribution matches that of the fragmented DNA. **(c)** Fragment-length distribution for the same DNA library analyzed by TGIRT-seq. The curves (3 technical replicates; EG1-3; see Supplementary Table S2 online) show a peak at 156 nt. Abbreviation: FU, fluorescence units.

Supplementary Figure S4

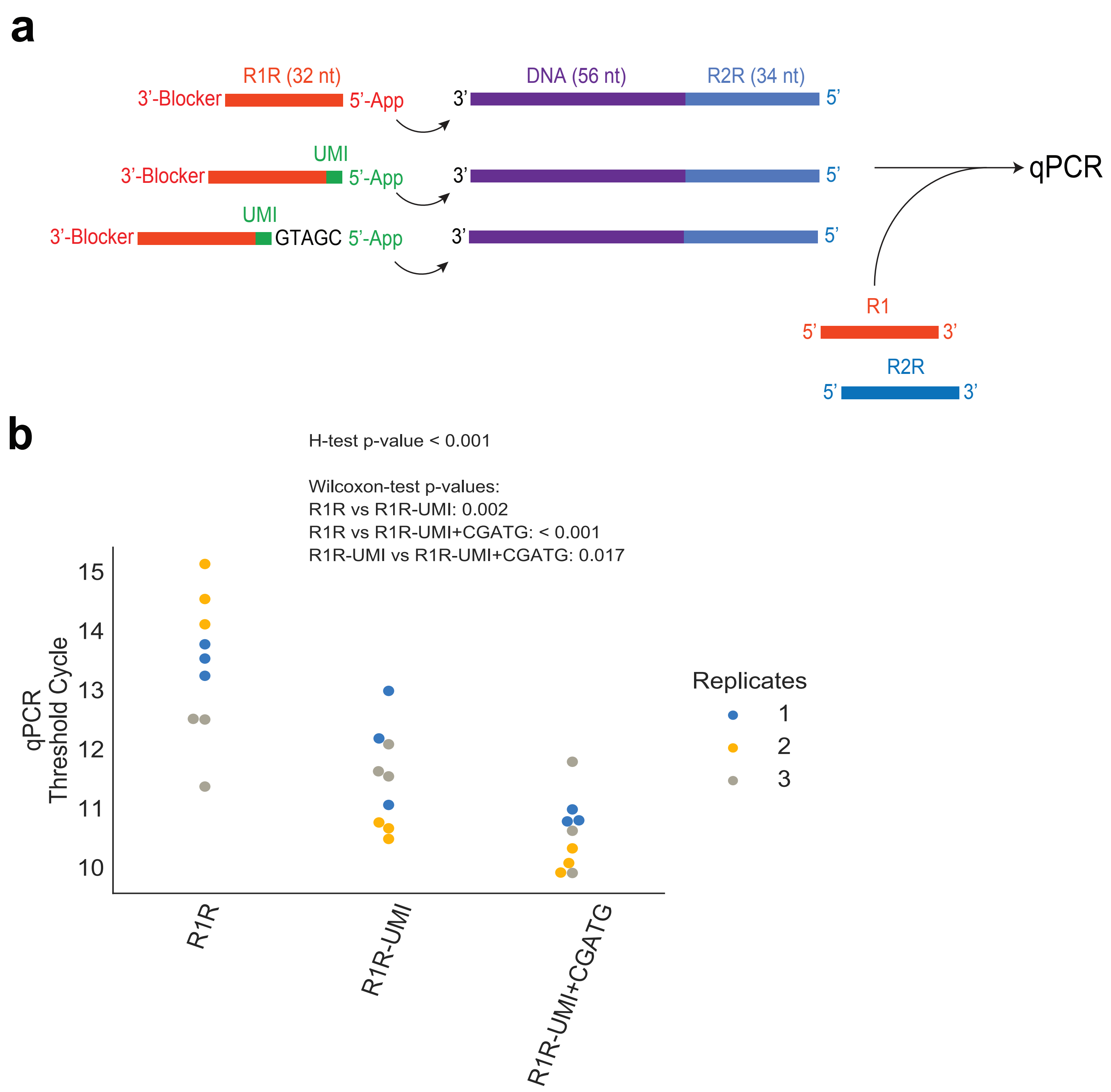

Supplementary Figure S4 / Ligation efficiencies of different 5' adenylated R1R DNA-seq adapters to a 90-nt DNA oligonucleotide. (a) Outline of the experiment. Three different versions of the R1R DNA without or with a 13-nt UMI directly at its 5' end or preceded by a short fixed sequence (CGATG) were ligated to a 90-nt DNA oligonucleotide containing an R2R primer-binding site by using Thermostable 5' AppDNA/RNA ligase (New England Bio- labs), according to the manufacturer's protocol. Ligation efficiency was measured by qPCR with R1 and R2R primers using a KAPA SYBR fast qPCR kit on a ViiA7 real-time PCR system (Thermo Fisher). **(b)** Threshold cycle of triplicate qPCR reactions from three different replicates of the ligation reactions (denoted 1, 2, and 3). The threshold cycles show a significant increase in ligation efficiency for the R1R oligonucleotides containing the UMI. p-values for Kruskal-Wallis H-test and Wilcoxon-Mann-Whitney U-test are indicated in the figure.

Supplementary Figure S5

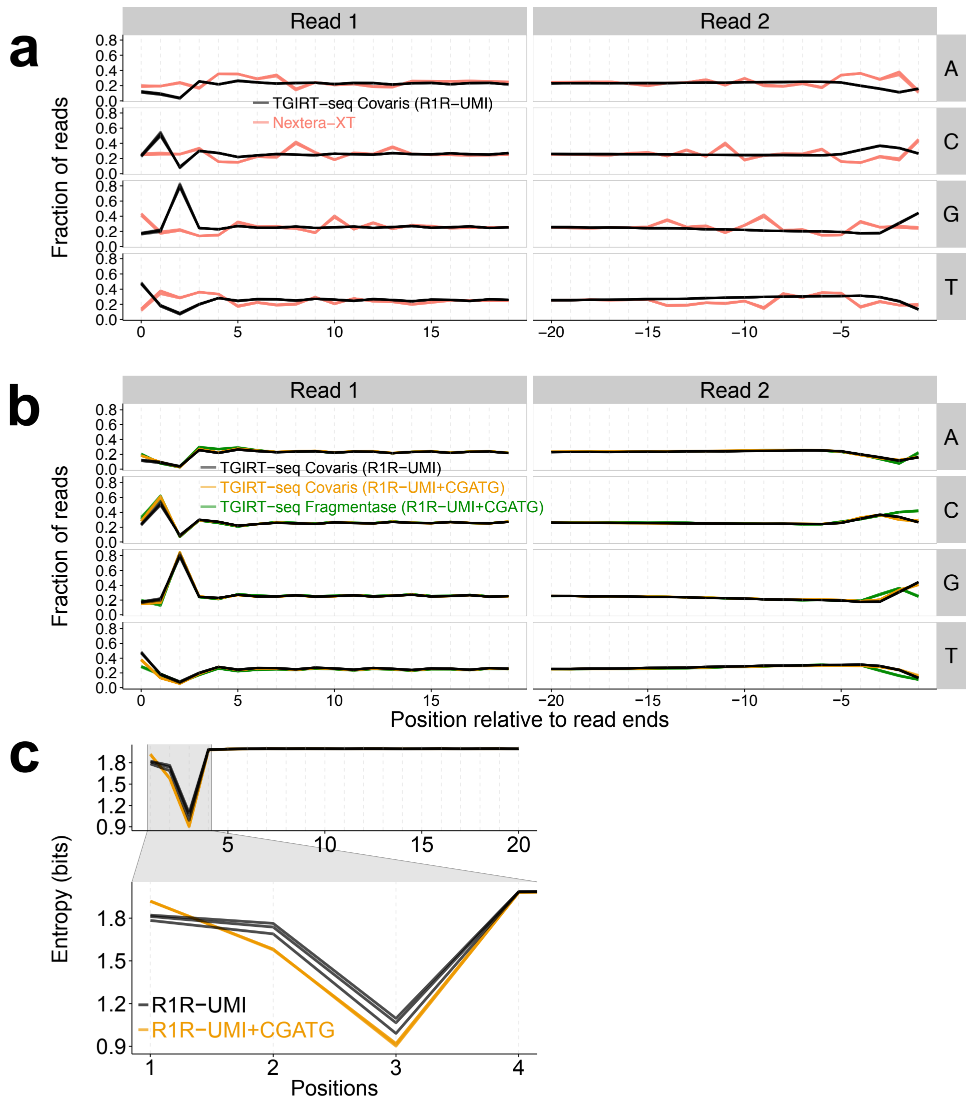

Supplementary Figure S5 / Analysis of 5'- and 3'-end biases in TGIRT-seq of *E. coli* genomic DNA with variations of the library preparation method. DNA-seq libraries were prepared from *E. coli* K12 strain MG1655 DNA that had been fragmented by different methods (Covaris sonication or Fragmentase; New England Biolabs) and/or using different R1R adapters (with a UMI either directly at its 5' end (R1R-UMI; datasets EG1-3) or preceded by a short fixed sequence (R1R-UMI+CGATG; datasets EG4-7; Supplementary Tables 1 and 2). Nucleotide compositions were extracted from the alignments by using a customized program. **(a)** 5'- and 3'-end sequence biases for TGIRT-seq compared to Nextera-XT. The plots show the fraction of different bases for the first 20 nucleotide positions at the 5' and 3' ends of the sequenced DNA fragment (5' ends of Read 1 and Read 2, respectively) for TGIRT-seq libraries prepared with DNA fragmented by Covaris sonication using R1R-UMI (black lines; 3 replicates; datasets EG1-3) compared to Nextera-XT (pink lines; 3 replicates). The plots show no substantial bias at the 3' end of the DNA fragment resulting from TGIRT template-switching, but do show bias for 5'-NCG at the 5' end of the DNA fragment, likely resulting from ssDNA ligation of the R1R adapter, as reported previously for RNA-seq[26]. **(b)** 5'- and 3'-end sequence biases for TGIRT-seq of *E. coli* genomic DNA fragmented by Covaris sonication using R1R-UMI (black lines; 3 replicates; same plots as panel (a)) or by Covaris sonication or Fragmentase using R1R-UMI+CGATG (2 replicates each; datasets EG4 and 5, orange lines, and datasets EG6 and 7, green lines, respectively). The results show that placing the 13-nt UMI directly at the 5' end of the R1R adapter reduces ligation bias at positions 2 and 3 of Read 1. **(c)** Close up of 5' end of Read 1 biases for Covaris-fragmented DNA with different R1R adapters from panel (b) quantified by information entropy. The R1R adapter with the UMI directly at its 5' end (R1R-UMI; black lines; 3 replicates) introduce less sequence bias as judged by higher sequence entropy at positions 2 and 3 than that in which the UMI is preceded by a short fixed sequence (CGATG; orange lines; 2 replicates).

Supplementary Figure S6

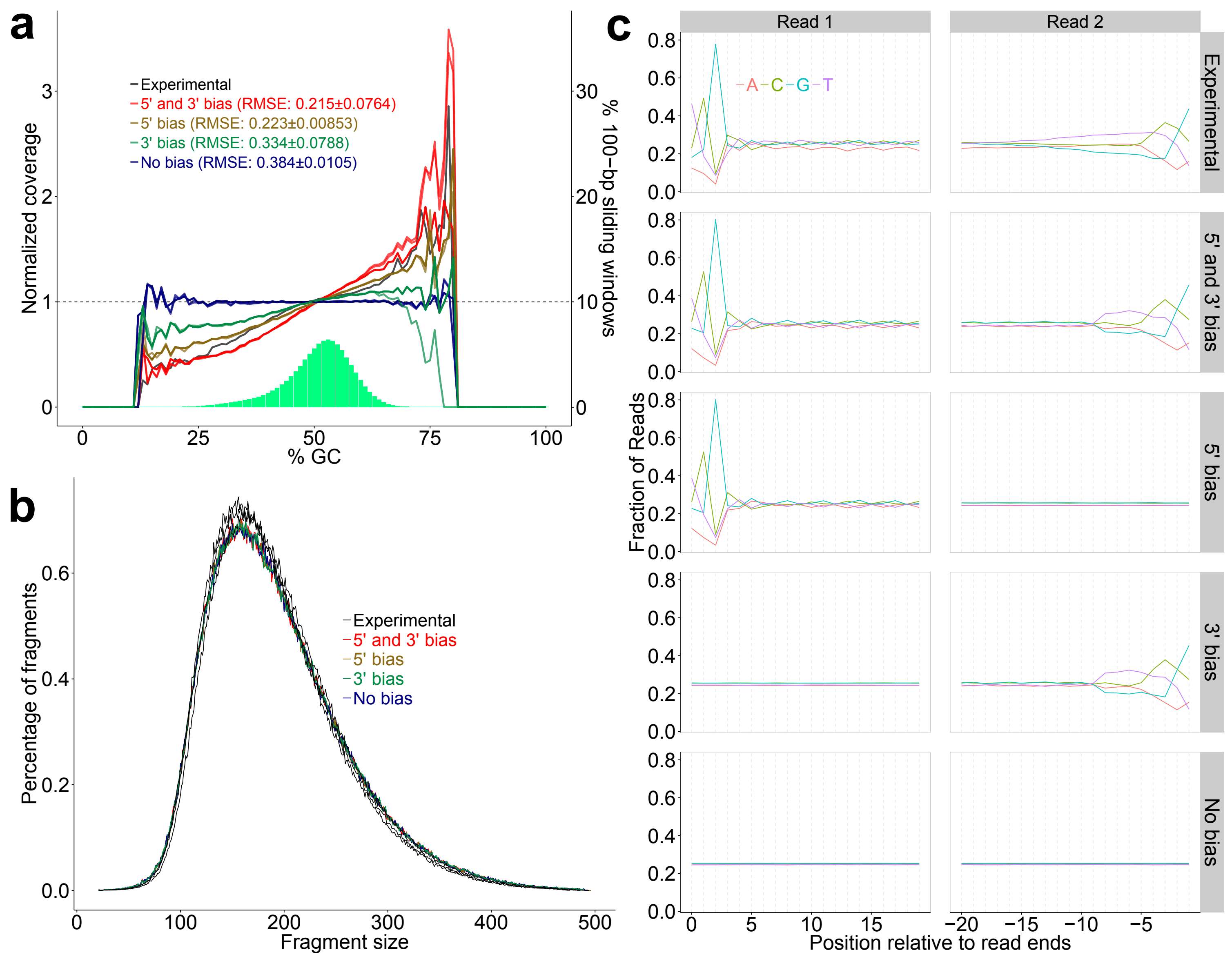

Supplementary Figure S6 / Simulations indicating that R1R ligation bias is the main source of the over-representation of GC-rich regions in TGIRT-seq of *E. coli* K12 genomic DNA. Simulated datasets were constructed by randomly sampling the *E. coli* K12 genome for sequences matching the fragment length distribution of the experimental TGIRT-seq dataset (EG1; see Supplementary Table S2 online) without (No bias) or with the experimentally determined 5'- and/or 3' end biases that resulted from using an R1R adapter with a 13-nt UMI directly at its 5' end (R1R-UMI) (see Supplementary Fig. S5 online). **(a)** Comparison of normalized coverage as a function of GC content calculated over a 100-nt sliding window for the experimental dataset and 5 simulated datasets (with 2 million fragments sampled for each dataset). The plots show that the simulated dataset generated by incorporating biases at both the 5'- and 3'-ends of the DNA fragment sequences for TGIRT-seq (red lines; root mean square error (RMSE) = 0.215) closely matches the experimental data (black line), and that most of the overrepresentation of GC-rich regions in the experimental data is due to ligation bias at the 5' end of the sequenced DNA fragment (gold lines; RMSE = 0.223). The green histogram shows the percentage of windows having different GC content across the *E. coli* genome. **(b)** Control showing that the fragment length distribution of the simulated dataset matches that of the experimental dataset. **(c)** Control showing that the 5'- and 3'-end biases for the simulated dataset match the experimental dataset for first 3 and 8 nts of Reads 1 and 2, respectively.

Supplementary Figure S7

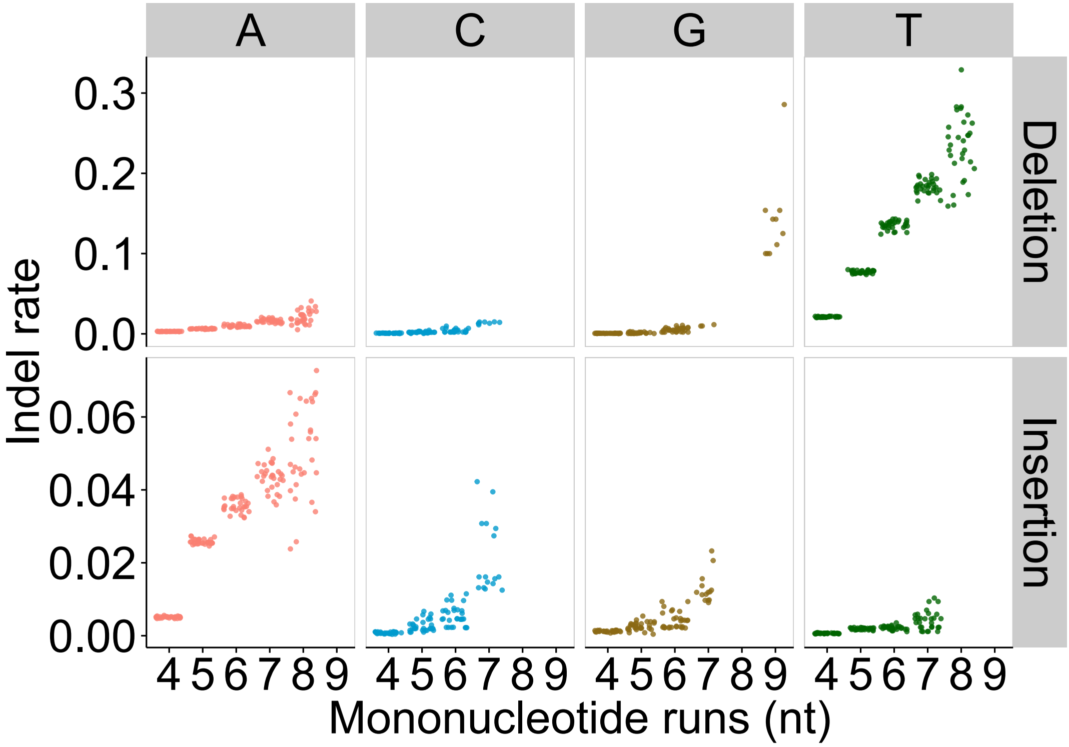

Supplementary Figure S7 / Indel rates for A, C, G, and T mononucleotide runs ≥4 nt. Each point represents one of ten subsampled libraries at 16X coverage from each of three TGIRT-seq technical replicates (datasets EG1-3; n = 30). The identity of the mononucleotide run that was the template for TGIRT-III was determined from the strand information of the mapped reads. The data show that TGIRT-seq is particularly prone to deletions at runs of T-residues and insertions at runs of A residues. In most cases, the insertions correspond to addition of one or two nucleotides of the same type as the mononucleotide run (A: 91.7 and 6.8%, respectively; C, 71.2 and 0.64%, respectively; G: 94.0 and 0.22%, respectively; T: 94.1 and 0.4%, respectively), suggesting that they result from template slippage during DNA synthesis.

Supplementary Figure S8

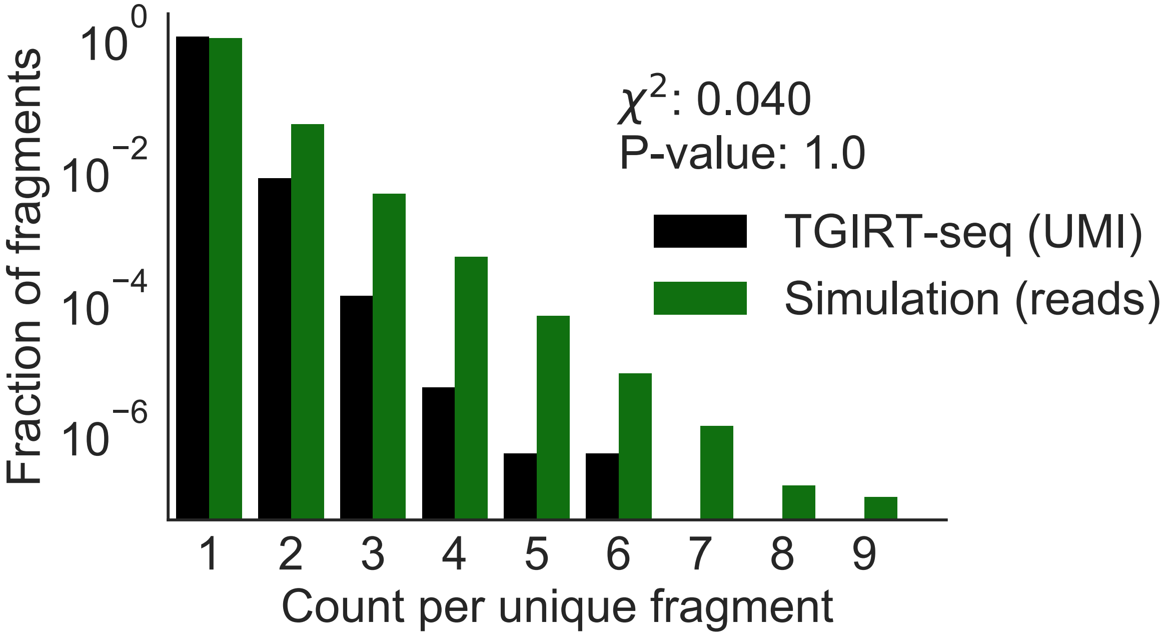

Supplementary Figure S8 / DNA template reutilization in TGIRT-seq. In the version of TGIRT-seq used here, the UMI is incorporated into the R1R DNA-seq adapter, which is ligated to the 3' end of the copied DNA stand (Fig. 1), and UMI-resampling could occur if a DNA template were copied more than once. To assess what proportion of DNA fragments might be copied twice or more, we determined how many UMI assignments correspond to unique or duplicate DNA fragments identified by 5'- and 3'-end genome coordinates, and we compared our experimental data (dataset EG1) to simulated data obtained by incorporating the DNA fragment 5'- and 3'-end biases found for TGIRT-seq (Supplementary Fig. 6). The latter estimates the number of identical fragments with different UMIs expected to result from biases in DNA fragmentation and ligation during library preparation. The experimental data were demultiplexed by tolerating up to 2 mismatches in the 13-nt UMI. The results show that the experimental and simulated data are statistically indistinguishable (Chi-square test p-value = 1), suggesting that minimal barcode resampling occurs during TGIRT-seq.

Supplementary Figure S9

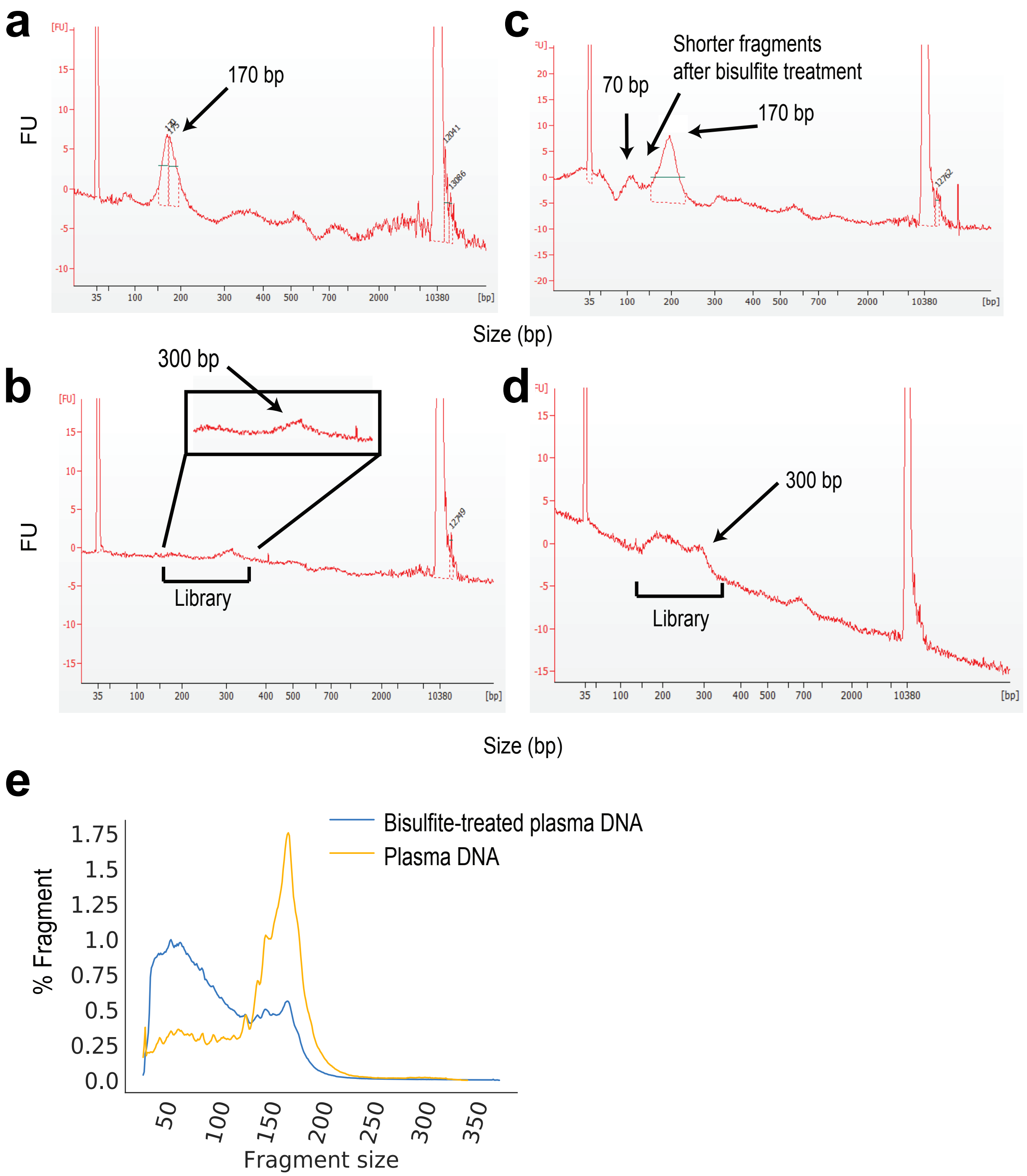

Supplementary Figure S9 / Bioanalyzer traces of plasma DNA, bisulfite-treated plasma DNA, and TGIRT-seq libraries constructed from these DNAs. (a) Input plasma DNA analyzed on an Agilent High Sensitivity DNA Bioanalyzer chip. The DNA was isolated from 3 ml of plasma obtained from a healthy male individual by using a Qiagen DSP DNA Blood Mini Kit and eluted with 100 µl of Qiagen buffer AE. 1 µl of the eluted product was analyzed on the Bioanalyzer. **(b)** TGIRT-seq library (1 µl of 30 µl of final library) constructed from 2 ng of plasma DNA analyzed on an Agilent High Sensitivity DNA Bioanalyzer chip. The library shows a peak at ~300 bp, corresponding to the peak size of plasma DNA (~170 bp) plus the DNA-seq adapters after PCR (3' adapter: P7 + 6 nt sample barcode + R2 = 64 nt; 5' adapter: P5 + R1 + UMI = 78 nt). **(c)** Bisulfite-treated plasma DNA (1 µl of 10 µl eluate after treatment with a Zymo EZ DNA Methylation-lightning kit) analyzed on an Agilent High Sensitivity DNA Bioanalyzer chip. Bisulfite-treatment results in an increased proportion of short DNA fragments compared to untreated DNA (panel a). **(d)** Final TIGRT-seq library (1 µl of a 30 µl solution constructed from 5 ng of bisulfite-treated DNA) analyzed on an Agilent High Sensitivity Bioanalyzer chip. The completed library shows more short DNA fragments, paralleling the increased proportion of short DNA fragments in the input DNA after bisulfite-treatment. **(e)** DNA-seq fragment-length distribution from TGIRT-seq libraries constructed from plasma DNA with and without bisulfite-treatment (datasets BPD1 and PD1, respectively). Abbreviation: FU, fluorescence units.

Supplementary Figure S10

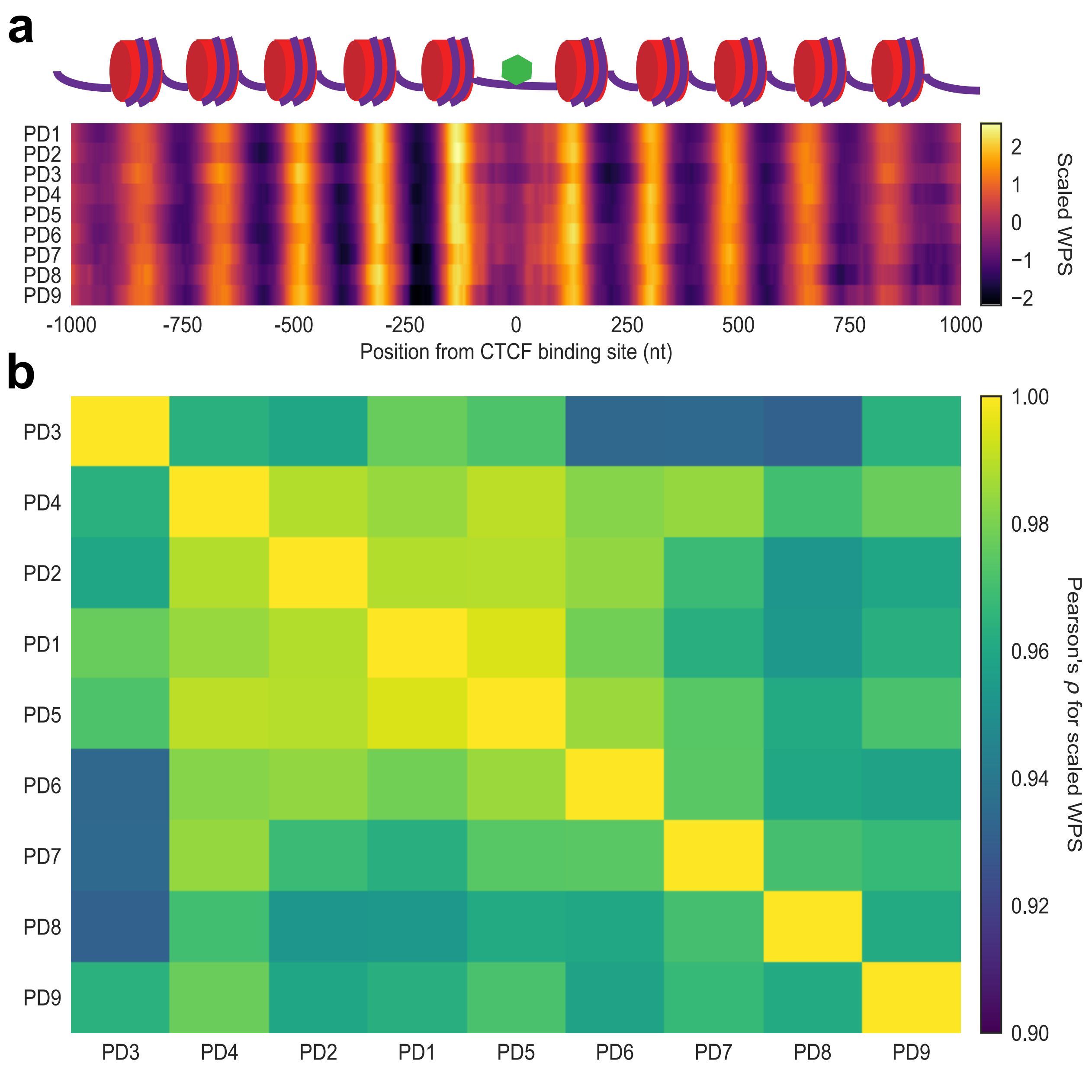

Supplementary Figure S10 / Reproducibility of nucleosomal positioning in individual TGIRT-seq datasets of plasma cfDNA from a healthy male individual. (a) Meta-analysis of scaled window protection scores (WPSs) within ±1 kb of a transcription factor CTCF binding site (green hexagon) in TGIRT-seq replicates. All 9 datasets (PD1-9) show strong nucleosome occupancies (high scaled WPSs; yellow) at similar positions around CTCF binding sites. **(b)** Pearson's correlation matrix of scaled WPSs. Pearson correlations were computed from the scaled WPSs in the datasets of panel a. High correlations (Pearson's correlation >0.93) indicate reproducibility of nucleosome positioning surrounding CTCF binding sites in the different TGIRT-seq datasets.

Supplementary Figure S11

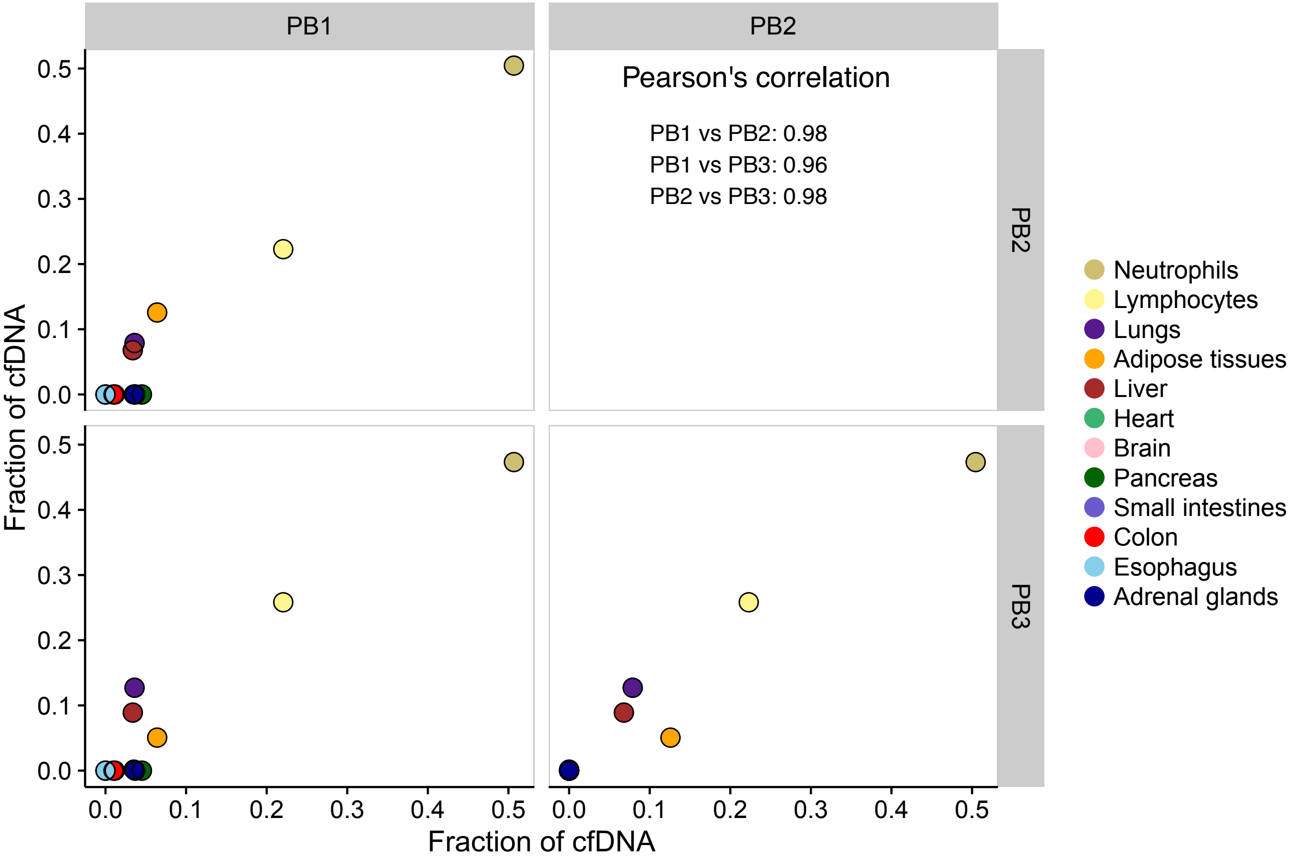

Supplementary Figure S11 / Reproducibility of TGIRT bisulfite sequencing of cfDNA. Tissue contributions determined from 3 replicates of TGIRT bisulfite sequencing of plasma cfDNA samples from a healthy male individual were plotted against each other and Pearson's correlations were computed. Pearson's correlation coefficients were 0.96 for each pairwise combination of the three individual datasets. All datasets indicate neutrophils (47-51%) and lymphocytes (22-26%) were the major contributors to cfDNA.
